# Optimization of the precursor supply for an enhanced FK506 production in *Streptomyces tsukubaensis*

**DOI:** 10.3389/fbioe.2022.1067467

**Published:** 2022-11-18

**Authors:** Susann Schulz, Christoph Schall, Thilo Stehle, Christian Breitmeyer, Sergii Krysenko, Agnieszka Mitulski, Wolfgang Wohlleben

**Affiliations:** ^1^ Department of Microbiology and Biotechnology, Interfaculty Institute of Microbiology and Infection Medicine Tübingen (IMIT), University of Tübingen, Tübingen, Germany; ^2^ Cluster of Excellence ‘Controlling Microbes to Fight Infections’, University of Tübingen, Tübingen, Germany; ^3^ Interfaculty Institute of Biochemistry, University of Tübingen, Tübingen, Germany

**Keywords:** pipecolic acid, FK506 production, tacrolimus, lysine cyclodeaminase, *S. tsukubaensis*

## Abstract

Tacrolimus (FK506) is a macrolide widely used as immunosuppressant to prevent transplant rejection. Synthetic production of FK506 is not efficient and costly, whereas the biosynthesis of FK506 is complex and the level produced by the wild type strain, *Streptomyces tsukubaensis*, is very low. We therefore engineered FK506 biosynthesis and the supply of the precursor L-lysine to generate strains with improved FK506 yield. To increase FK506 production, first the intracellular supply of the essential precursor lysine was improved in the native host *S. tsukubaensis* NRRL 18488 by engineering the lysine biosynthetic pathway. Therefore, a feedback deregulated aspartate kinase AskSt* of *S. tsukubaensis* was generated by site directed mutagenesis. Whereas overexpression of AskSt* resulted only in a 17% increase in FK506 yield, heterologous overexpression of a feedback deregulated AskCg* from *Corynebacterium glutamicum* was proven to be more efficient. Combined overexpression of AskCg* and DapASt, showed a strong enhancement of the intracellular lysine pool following increase in the yield by approximately 73% compared to the wild type. Lysine is coverted into the FK506 building block pipecolate by the lysine cyclodeaminase FkbL. Construction of a *∆fkbL* mutant led to a complete abolishment of the FK506 production, confirming the indispensability of this enzyme for FK506 production. Chemical complementation of the *∆fkbL* mutant by feeding pipecolic acid and genetic complementation with *fkbL* as well as with other lysine cyclodeaminase genes (*pipAf, pipASt*, originating from *Actinoplanes friuliensis* and *Streptomyces pristinaespiralis*, respectively) completely restored FK506 production. Subsequently, FK506 production was enchanced by heterologous overexpression of PipAf and PipASp in *S. tsukubaensis*. This resulted in a yield increase by 65% compared to the WT in the presence of PipAf from *A. friuliensis*. For further rational yield improvement, the crystal structure of PipAf from *A. friuliensis* was determined at 1.3 Å resolution with the cofactor NADH bound and at 1.4 Å with its substrate lysine. Based on the structure the Ile91 residue was replaced by Val91 in PipAf, which resulted in an overall increase of FK506 production by approx. 100% compared to the WT.

## Introduction

Tacrolimus (FK506) is a 23-membered macrocyclic polyketide that has firstly been isolated from *Streptomyces tsukubaensis* in 1984 (Kino et al., 1987). It exhibits a strong immunosuppressive activity by blocking the calcineurin phosphatase leading to a reduced T-cell proliferation due to the diminished level of interleukin-2, an essential growth factor for activated T-cells (Schreiber & Crabtree, 1992). Although sharing a similar mode of action, FK506 is much more potent than the well-established immunosuppressant ciclosporin (Liu et al., 1991; Jiang & Kobayashi, 1999), hence arising great pharmaceutical interest (Haddad et al., 2006; Muduma et al., 2016). Nowadays FK506 is chosen as a first line drug in various clinical areas of application e.g., after organ transplantation or for treatment of inflammatory skin diseases and eczema leading to an emerging commercial and scientific interest (Staatz & Tett, 2004; Lin, 2010). Analysis of randomized controlled trials showed FK506 to be superior to ciclosporin in terms of patient mortality and hypertension (Muduma et al., 2016). However, the biotechnological production is limited due to the low titers of FK506 produced by the wild type strains. While some groups focused on classical feeding strategies by supplementing relevant precursors and media optimization (Xia et al., 2013; Martínez-Castro et al., 2013; Ban et al., 2016), others tried to solve the problem by genetic manipulation targeting metabolic pathways, e.g., *via* overexpression of FK506 biosynthetic genes (Huang et al., 2013; Ban et al., 2016; Fu et al., 2016; Ordóñez-Robles et al., 2018).

**FIGURE 1:**
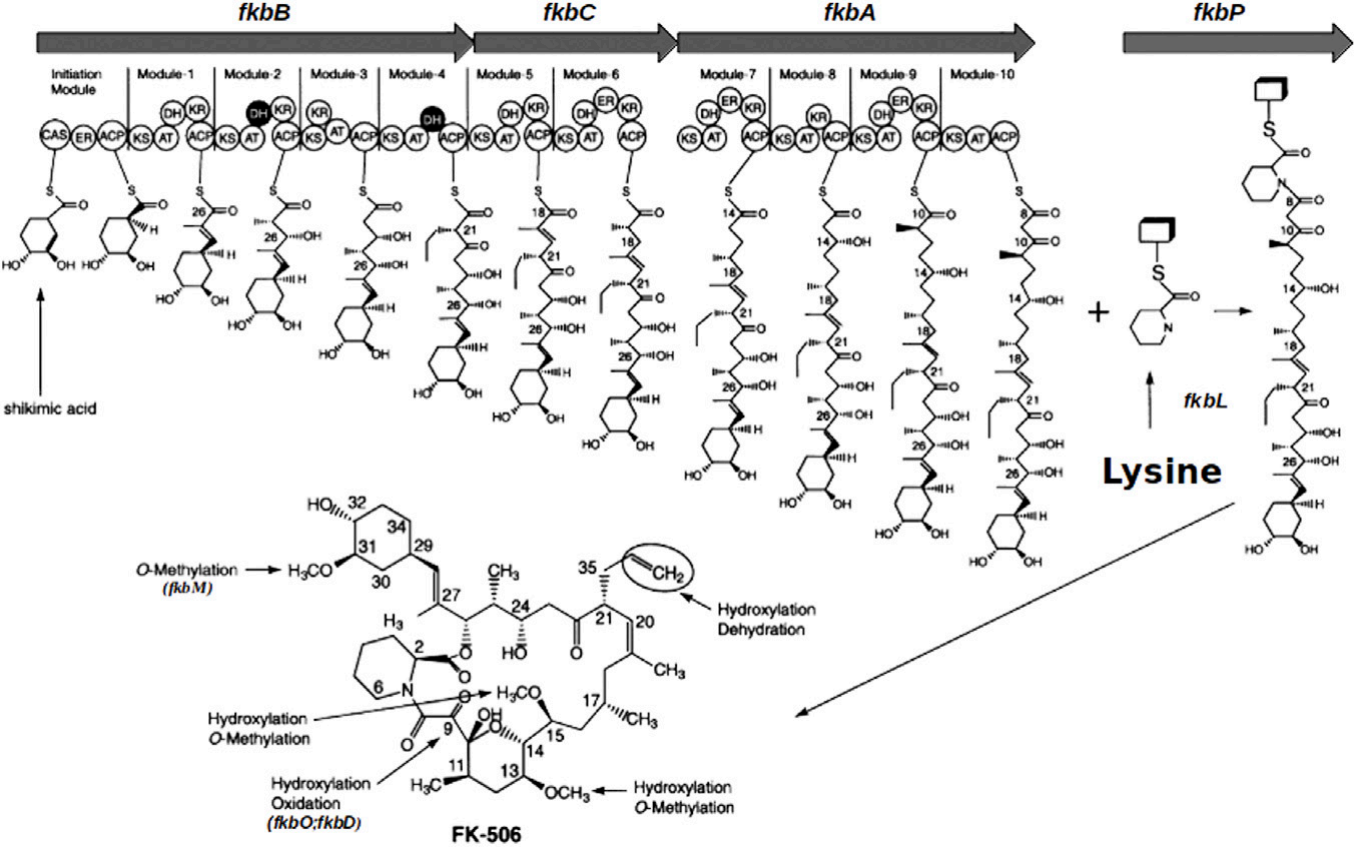
Schematic representation of the FK506 biosynthesis in *S. tsukubaensis* (modified after Ordóñez-Robles et al., 2018). Arrows in the upper part: three PKS genes (*fkbA*, *fkbB*, *fkbC*) and the NRPS gene (*fkbP*) of the biosynthetic cluster. Boxes M1 to M10: Modules of the PKSs; circles are domains in the modules: ACP, acyl carrier protein; AT, acyltransferase; ER, enoyl reductase; CAS, CoA synthetase; KR, 3-oxoacyl (ACP) reductase; DH, 3-oxoacyl thioester dehydratase; KS, 3-oxoacyl (ACP) synthase. DHCHC: (4R, 5R)-4,5-dihydroxycyclohex-1-enecarboxylic acid.

The biosynthetic pathway of FK506 has been entirely elucidated (Motamedi et al., 1998) and the whole genome sequencing of the FK506 producer *S. tsukubaensis* NRRL 18488 has been completed (Barreiro et al., 2012). Furthermore, modular polyketide synthase and nonribosomal peptide synthetase genes have been described in this strain (Blazic et al., 2012). Basically, the biosynthesis of the core polyketide of FK506 is processed by three different polyketide synthases (PKS) FkbA, FkbB and FkbC, catalyzing the condensation of an unusual starter unit derived from the shikimic acid pathway (4,5-dihydroxycyclo-1-enecarboxylic acid (DHCHC)) with ten extender units (two malonyl-CoA, five methylmalonyl-CoA and two methoxymalonyl-CoA). In a following step the non-ribosomal peptide synthetase (NRPS) FkbP is incorporating the sole peptide moiety pipecolic acid, which is derived from L-lysine, into the polyketide chain. It closes the ring structure in a final cyclization step (Motamedi et al., 1997; Motamedi & Shafiee, 1998; Goranovic et al., 2010; Andexer et al., 2011; Mo et al., 2011) before the immature macrolactone is further processed by post-PKS tailoring enzymes resulting in the final compound FK506 (Motamedi et al., 1996; Chen et al., 2013) (Figure 1). Pipecolic acid, a non-proteinogenic amino acid, is a key intermediate in the synthesis of a large number of drugs, e.g., pristinamycin (Mast et al., 2011), friulimicin (Müller et al., 2007), meridamycin (Jiang et al., 2011), rapamycin (Gatto et al., 2006), tacrolimus (Turlo et al., 2012), nocardiospin (Bis et al., 2015), tubulysin B (Steinmetz et al., 2004) and others. In fact, pipecolic acid is often even crucial for the bioactivity of secondary metabolites (Min, 2018).

**FIGURE 2:**
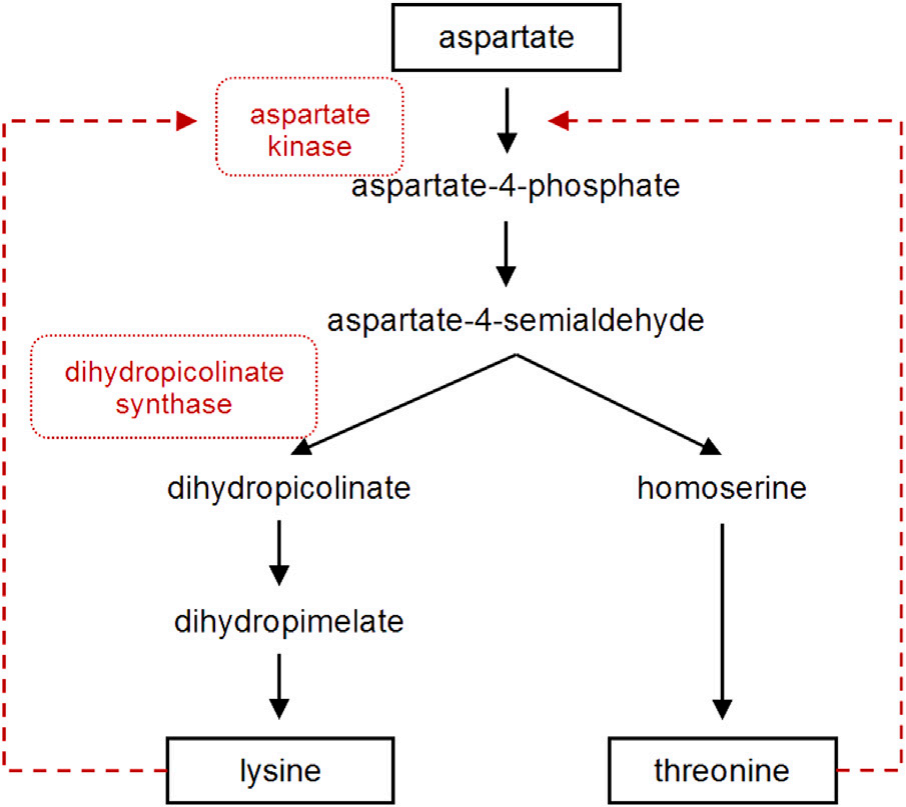
The postulated lysine and threonine biosynthesis pathway in *S. tsukubaensis*. Dashed arrows represent feedback inhibition. Enzymes that were overexpressed in this study are marked in red.

The biosynthesis of lysine has extensively been studied in microorganisms leading to a biotechnological production in high level producer strains like *C. glutamicum* and *E. coli* (Sahm et al., 1996). In *E. coli*, the combined plasmid-based overexpression of *dapA*, *lysA* and *lysC* from *E. coli* under the control of the strong trc promoter as well as the overexpression of ddh from *C. glutamicum* were studied for their role in the lysine biosynthesis to increase the biosynthetic pool of L-lysine (Ying et al., 2017). But this pathway has not specifically been optimized for the production of pipecolate containing metabolites in actinobacteria, taxonomically related to corynebacteria. Studies in *C. glutamicum* provided good understanding of the lysine biosynthesis. This process includes two central optimization steps involving the dihydropicolinate synthase DapA, which is the branching point between lysine and threonine pathway, and the aspartate kinase Ask, which catalyzes the first step in this biosynthetic pathway and is strictly regulated by its end products lysine and threonine (Figure 2). It has been shown that high-level lysine producers of *C. glutamicum* possess a defect in feedback inhibition of the corresponding aspartate kinases (Pérez-García et al., 2016).

Pipecolic acid represents a lysine derived, non-proteinogenic amino acid embedded in the polyketide core structure of the FK506 molecule. In some microorganisms, the biosynthesis of the pipecolic acid has been described to occur *via* two-step biosynthesis routes (Gupta & Spenser, 1969; He, 2006; Tani et al., 2015). In contrast, during the biosynthesis of rapamycin in S. hygroscopicus the conversion of L-lysine to L-pipecolic acid in one step by a lysine cyclodeaminase (LCD) has been shown to be directly catalyzed (Gatto et al., 2006). The one-step pipecolic acid synthesis *via* the LCD has the advantage to be more suitable for combining it with upstream metabolic processes (Gatto et al., 2006). For example, it was reported that a recombinant *E. coli* strain overexpressing LCD *pipASp* could produce L-pipecolic acid from L-lysine with a yield increase of nearly 70% compared to the parental strain *E. coli* BL21 (DE3) (Ying et al., 2015).

To ensure the supply of the pipecolic acid in S. tsukubaensis, the FK506 biosynthetic gene cluster contains the gene *fkbL*. Due to the similarity of FkbL to ornithine cyclodeaminases, the product of *fkbL* is predicted to be a LCD that catalyzes the conversion of L-lysine to pipecolic acid in a deaminative cyclization reaction. The resulting non-proteinogenic amino acid is then activated and attached to the polyketide chain by the NRPS FkbP. The pipecolate moiety plays a crucial role for the biological activity of FK506 as it is part of the binding motif for the cognate immunophilin (FKBP-12) in T-cells (Andexer et al., 2011).

Lately a lot of efforts have been invested in optimizing the biotechnological process of FK506 fermentation by employing different strategies ranging from classical strain improvement methods to more purposeful metabolic profiling approaches (Yoon & Choi, 1997; Xia et al., 2013; Ban et al., 2016). Since the major bottleneck for a high FK506 yield may be the limited supply of building blocks, plenty of the scientific work on the FK506 production improvement was dedicated to the optimization of increased levels of precursors. Most strategies focused on specific supplementation of the production media with essential precursors. Others directly applied genetic approaches for the targeted engineering of biosynthetic pathways which deliver FK506 building blocks (Chen et al., 2012; Turło et al., 2012; Huang et al., 2013; Huang et al., 2013). Particularly, an effect of L-lysine on the production of FK506 as well as other antibiotics, like ß-lactams, has already been shown in previous feeding studies in *Streptomyces sp.* (Mendelovitz and Aharonowitz; 1982; Mendelovitz and Aharonowitz; 1983; Martínez-Castro et al., 2013), *Escherichia coli* (Ying et al., 2017) as well as in such Actinobacteria as *Amycolatopsis lactamdurans* (Hernándo-Rico et al., 2001).

In last years, an efficient strategy for heterologous *de novo* biosynthesis of the FK506 intermediate L-pipecolic acid was presented in recombinant *E. coli* cells (Ying et al., 2017). In order to enhance the metabolic pathway from L-lysine to L-pipecolic acid, *pipA*, a lysine cyclodeaminase gene from *S. pristinaespiralis* was introduced. Additional plasmid-based overexpression of *dapA*, *lysC*, and *lysA* under the control of the strong trc promoter and rebalancing of the intracellular pyridine nucleotide concentration increased the pipecolic acid production (Ying et al., 2017). In this work a new optimization strategy that interconnects the enhancement of the precursor supply for the primary pathway of the L-lysine biogenesis with the secondary pathway of pipecolic acid formation in the native host *S. tsukubaensis* is presented. Among the few routes that have evolved to generate pipecolic acid, the lysine cyclic deamination pathway was mainly found in actinobacteria. The FK506 gene cluster as well as the pristinamycin and friulimicin gene clusters comprise a gene encoding a lysine cyclodeaminase (*fkbL*, *pipASt* and *pipAf*, respectively). This type of enzyme was biochemically characterized and the crystal structure of PipA from *S. pristinaespiralis* has been solved recently (Ying & Chen, 2016).

In order to optimize the precursor flux we aimed to combine optimization of specific natural product synthesis in the endogenous system as well as the synthetic biology approach with genes from bacteria that are used for the biotechnological production of amino acids. Combining the information deduced from the pipecolic acid biogenesis from different microorganisms and from the deposited crystal structure of the lysine cyclodeaminase PipASp as well as in this work determined structure of PipAf, a unique approach for the enhancement of FK506 production in *S. tsukubaensis* had been achieved.

## Materials and methods

### Bacterial strains and plasmids

*S. tsukubaensis* NRRL 18488 from the NRRL Culture Collection of the Agricultural Research Service (USA). All mutants are derivatives of *S. tsukubaensis* NRRL 18488. *Escherichia coli* Novablue cells from Novagen were used for standard cloning procedures, while *E. coli* Rosetta DE3 cells served as hosts for protein expression experiments. For plasmid transfer intergeneric conjugation between the non-methylating *E. coli* ET12567/pUZ8002 strain and *S. tsukubaensis* was used (Kieser et al., 2000). For overexpression experiments the integrative pRM4 vector containing the strong constitutive ermE promoter was used (Menges et al., 2007).

For heterologous expression experiments concerning the lysine cyclodeaminases, the *pipA* gene was amplified from genomic DNA from *Streptomyces pristinaespiralis* Pr 11 (Aventis Pharma) (Mast et al., 2011). As template for the amplification of the *pip* gene from *Actinoplanes friuliensis* the plasmid pRF37 from C. Müller et al., 2007 was used (Müller et al., 2007). *S. tsukubaensis* NRRL 18488 was used as a template for the amplification of the *fkbL*, *fkbP* and *dapA*.

The genomic DNA for the amplification of the feedback inhibition deregulated aspartate kinase gene lysCCg* from *Corynebacterium glutamicum* DM1730 (Seibold et al., 2006) was kindly provided by Jörn Kalinowski (University of Bielefeld, Germany).

All strains and plasmids used in this study are listed in the Supplementary Table S2. All oligonucleotides used in this work are listen in the Supplementary Table S3.

### Generation of the *S. tsukubaensis ΔfkbL* mutant

For generation of the *S. tsukubaensis fkbL* mutant, a 2.2 kb upstream and downstream regions of the *fkbL* gene were amplified from the genomic DNA of *S. tsukubaensis* NRRL 18488 with the primer pair fkbLupfw/fkbLuprev and fkbLdownfw/fkbLdownrev. The apramycin resistance cassette was amplified from the vector pSET152 using the primers aprneufw/aprneurev. Purified PCR fragments have been first cloned in the intermediate vector pDrive and afterwards in the pK18 vector. Novablue competent *E. coli* cells were then transformed with the recombinant plasmid pK18oriT*ΔfkbLaprR* containing the inserted *fkbL* upstream and downstream fragments as well as the apramycin resistance cassette. The clones were selected for kanamycin and apramycin resistance and verified by sequencing. Afterwards, the pK18oriT*ΔfkbLaprR* plasmid was transferred into the methylation-deficient *E. coli* ET12567/pUZ8002 and *E. coli* ET12567/pUB307 strain. After the biparental conjugation between the methylation-deficient *E. coli* strains and *S. tsukubaensis* NRRL 18488, the ex-conjugants were selected for the double recombination event at both flanking homologous sequences by demonstrating apramycin resistance and kanamycin sensitivity.

### Media and culture conditions

Spores and mycelia preparations were obtained from ISP4 (Difco, Sparks, MD, USA) agar plates. FK506 production by *S. tsukubaensis* NRRL 18488 was analyzed in the liquid media MG optimized by Martínez-Castro et al., 2013. FK506 production was also analyzed in R2YE medium (Kieser et al., 2000) modified by Temuujin et al., 2011. The composition of MGm medium was as follows: 50 g/l starch (Difco), 8.83 g/l glutamic acid, 2.5 mM KH2PO4/K2HPO4, 0.2 g/l MgSO4·7H2O, 1 mg/l CaCl2, 1 mg/l NaCl, 9 mg/l FeSO4 ·7H2O, 21 g/l MOPS, and 0.45 ml/l 10× trace elements, adjusted to pH 6.5. The composition of R2YE medium was as follows: 103 g/l saccharose, 0.25 g/l K2SO4, 10.12 g/l MgCl2*6H2O, 10 g/l glucose, 0.1 g/l casamino acids, 5 g/l yeast extract, 10 ml/l 0.5% K2HPO4, 80 ml/l 3.68% CaCl2*2H2O, 15 ml/l 20% L-prolin, 100 ml/l 5.73% TES, 2 ml/l trace elements, pH 7,2. For 10 ml solution (100×) of trace elements, the components are: 39.0 mg CuSO4 ·5H2O, 5.7 mg H3BO3, 3.7 mg (NH4)6Mo7O24·4H2O, 6.1 mg MnSO4·H2O, and 895.0 mg ZnSO4·7H2O. Fermentation was performed using a two-stage culture system. For the seed culture a mixture of 1:1 modified YEME (3 g/l yeast extract, 5 g/l Bacto peptone, 3 g/l malt extract, 10 g/l glucose and 340 g/l sucrose) and Tryptic Soy Broth (Difco) was inoculated with spores or mycelia from an ISP4 plate and cultivated for at least 3 days at 28°C and 220 rpm. For the main culture 100 ml of MGm medium were inoculated with the seed culture and further incubated at the same condition for 6 days.

### Analysis of growth and FK506 production

The productivity of an *S. tsukubaensis* strain was usually determined as production of FK506 per g biomass (cell dry weight). During the fermentation time of 6 days all 24 h 1 ml of bacterial culture was gathered, centrifuged, washed twice with destilated water, and dried by lyophillization. For FK506 determination, the broth samples were mixed with equal volume of ethylacetate (1:1), stirred for 10 min and subsequently centrifuged. The organic phase was analysed *via* an HP1090 M HPLC system equipped with a diode array detector and a thermostatic autosampler. A Zorbax Bonus RP column, 3 × 150, 5 μm (Agilent Technologies, Santa Clara, USA) constituted the stationary phase. The mobile phase system was applied with 0.1% phosphoric acid and 0.2% triethylamine as eluent A and acetonitrile with 1% tetrahydrofurane as eluent B. The flow rate was 850 μL/min, the column temperature was set to 60°C. The absorbance was monitored at a wavelength of 210 nm. Data sets were processed with the help of the Chemstation LC 3D, Rev. A.08.03 software from Agilent Technologies. Standards of pure FK506 (Antibioticos SA) and ascomycin (Sigma-Aldrich Chemie GmbH, Munich, Germany) were used as controls.

### Homology protein modelling and analysis of protein 3D structures

A 3D protein model of a target sequence was generated in comparative modelling by extrapolating experimental information from an evolutionary related protein structure that serves as a template using SWISS-MODEL (Waterhouse et al., 2018). Template search was based on the target sequence, which served as a query to search for evolutionary related amino acid sequences by BLASTP (Altschul et al., 1997) and for protein structures against the SWISS-MODEL template library. Templates were ranked according to expected quality of the resulting models and used to automatically generate 3D protein models by loop modelling. The quality of obtained models was estimated based on expected model accuracy, the QMEAN scoring function (Studer et al., 2020) as well as based on the Ramachandran plot (Ramachandran et al., 1963). 3D models of proteins were analyzed using the Swiss-PdbViewer software (Guex et al., 2009).

### Expression and purification of the his-tagged Pip protein

The *pipAf* gene was amplified using the above mentioned template from Müller et al., 2007. It was cloned in the expression vector pET30 Ek/lic containing an N-terminal His-tag with the help of the ligation independent Ek/lic system according to the protocol proposed by Novagen, UK. E.*coli* Rosetta DE3 was transformed with the resulting construct pET30/*pip*. 200 ml of Luria Bertami broth (LB) (Sambrook & Russell, 2006) with kanamycin (50 μg/ml) and chloramphenicol (34 μg/ml) was inoculated with 2 ml of an overnight culture and incubated at 37°C on a rotary shaker (180 rpm). Expression was induced with 1 mM IPTG after the culture broth has reached an OD578 of 0.4. The induced culture was further incubated for 18 h. The cells were harvested by centrifugation, resuspended in lysis buffer (20 mM Tris, 100 mM NaCl, 20 mM imidazol, protease inhibitor (Roche, Mannheim, Germany)) and lysed by 3-4 passages through Emulsiflex B15 (Avestin, Ottawa, Canada). Protein purification was either carried out by gravity flow Ni NTA Superflow columns (Iba) or FPLC using a His-TrapFF 1 ml column (GE Healthcare, Munich, Germany). Concentrated protein eluates were stored at 4°C.

### Construction and expression of Pip* variants

Mutated Pip* variants were generated by site-directed mutagenesis. The procedure was based on the Stratagene quickchange protocol, which was optimized by Zheng et al. i (2004). For each mutation specific primers were designed. Mutations were introduced into the pET30/*pip* expression plasmid using PCR. Non-mutated template DNA was digested using the restriction enzyme DpnI. Subsequently, the competent *E. coli* Novablue strain was transformed. Plasmid-carrying clones were identified by selection for kanamycin and verified by PCR and sequencing.

### Crystallization and structure determination of Pip

The obtained PipAf was concentrated by Amicon Ultra spin concentrators and further purified by gel filtration on a Superdex 200 run with 150 mM NaCl and 25 mM HEPES pH 7.4. The main peak corresponding to a dimer was pooled and concentrated up to absorption at 280 nm of 2.5 equal to a protein concentration of 9.5 mg/ml. The absorption at 340 nm thereof varied from 0.4 to 0.8 depending on the batch. For crystallization the concentrate was supplemented with 1 mM NAD+ and diluted to 7.3 mg/ml. The same solution was also used for co-crystallization with the substrate but contained additionally lysine to a final concentration of 140 mM. The crystallization drops contained 450 nl of protein and 300 nl of crystallization solution and were set up in sitting drop microtiterplates with a Tecan Freedom Evo pipetting robot. First crystals grew after few hours at 4°C in several conditions. Lysine-Cocrystallization was optimized with JCSG + suite screen (Qiagen, Hilden, Germany) condition 42 (20% PEG8000, 0.2 M MgCl2, 0.1 M Tris pH 8.5) and 10% glycerol added before freezing. From the setups without lysine crystals were obtained from condition 36 of Morpheus screen (Molecular dimensions) which were of sufficient quality.

The crystals were mounted in loops and flash-frozen in liquid nitrogen for storage and measured at the Swiss Light Source (SLS, Villingen, Germany). Data reduction was carried out with the XDS package. For phase determination with PHASER the OCD structures were truncated and used as search model. The structure was built in interactive cycles with coot and for refinement; simulated annealing was included in the very first refinement run. The representation of the solved protein structure was carried out using the free graphics 3D software PyMOL (The PyMOL Molecular Graphics System, Version 2.0 Schrödinger, LLC).

### Thin layer chromatography for qualitative detection of pipecolic acid

Overnight cultures of expression strains *E. coli* Rosetta 2 (pET/*pipAf*) and *E. coli* Rosetta 2 (pET/pipAf*) and the control *E. coli* Rosetta 2 were incubated overnight at 37°C in 10 ml LB medium with the corresponding kanamycin and chloramphenicol concentrations. Subsequently, 100 ml of LBKM/CM of the main culture was supplemented with 1 µL of the corresponding pre-culture and incubated up to an OD578 of 0.6 at 37°C. After reaching the OD578 of 0.6, the expression of the PipAf/PipAf* proteins was induced by adding 1 mM IPTG with the subsequent incubation at 28°C for further 20 h. At the next day, the main cultures were supplemented with 0.5 ml of the glycerol stock solution (50%) as well as 0.5 ml lysine stock solution (50%) and incubated for 6 h at 32°C. Afterwards, the cultures were centrifuged for 10 min at 5,000 rpm. To determine the formation of pipecolic acid, 5 µL of supernatant from each culture was spotted at a silica gel plate. The mobile phase, which consisted of a mixture of 3:1:1 N-butanol:acetic acid:water, was used for the separation of substances. As controls the pure lysine and pipecolic acid (5 µg each) were used. After the running front had reached a height of approx. 10 cm, the silica gel plate was stained with 0.5% ninhydrin solution and analyzed. Quantification of pipecolic acid on TLC was performed using the open-source software ImageJ: percentage of each peak was related to the total area of all peaks and represents the relative amount of the pipecolate based on digital imaging.

## Results and discussion

### Improvement of the lysine precursor supply in *Streptomyces tsukubaensis*

L-lysine is an essential amino acid, used in nutrition, as supplementary and nutraceutical, which production has been established in *C. glutamicum*. Also, lysine is an important product that serves as a precursor for pipecolic acid (piperidine-2 carboxylic acid), which is a non-proteinogenic amino acid widely used in all phylogenetic domains of life and which is a building block of biologically active substances such as streptogramin pristinamycin (Mast et al., 2011) as well as macrocyclic immunomodulators such as rapamycin and FK506 (Turlo et al., 2012). Enhancing the L-lysine precursor pool should result in a positive effect on secondary metabolite production. To proof this hypothesis classical media supplementation experiments with various amino acids have been performed showing that particularly exogenous L-lysine feeding lead to a significant boost of rapamycin (Cheng et al., 1995) respectively FK506 production (Huang et al., 2013; Martínez-Castro et al., 2013), consequently making the L-lysine primary biosynthesis pathway a putative target for genetic engineering. For instance, enrichment of the semi-defined medium for FK506 production in *S. tsukubaensis* with 2.5 g/l L-lysine enhanced the production by approximately 30% (Martínez-Castro et al., 2013). In order to test, whether additional lysine can further improve the FK506 yield, we extended this experiment by increasing the exogenous lysine end concentration beyond 2.5 g/l (17 mM). However, at higher exogenous lysine concentration (50 mM) the FK506 production in the wild type was clearly inhibited (Supplementary Figure S1), what can be attributed to feedback inhibition.

In order to avoid this feedback inhibition we intended to adjust the lysine biosynthetic pathway in *S. tsukubaensis* NRRL 18488 by overcoming the inhibition of the aspartate kinase and redirecting the carbon flux towards the lysine branch. We therefore chose the dihydropicolinate synthase (DapASt) and the aspartate kinase (AskSt) to be processed to direct the precursor flux towards lysine and hence towards elevated levels of the building block pipecolic acid.

The FK506 production in all strains grown in the MG production medium was analyzed by HPLC, whereby samples were taken after every 24 h and used for the biomass estimation and FK506 analytic. We analyzed the production after 6 days of *Streptomyces tsukubaensis* fermentation for two reasons. On the one hand, this strain is in the late stationary phase and the maximum biomass accumulation was achieved. On the other hand, many reports describe production of FK506 and other secondary metabolites in Actonibacteria in the late stationary phase as well (after 6–8 days of fermentation) (Müller et al., 2007; Jung et al., 2009; Goranovic et al., 2010; Mo et al., 2011; Chen et al., 2012).

In the WT strain the FK506 production in the MGm medium remained almost unchanged after 2 days of production (Supplementary Figure S2). Interestingly, although the WT demonstrated in our experiments higher initial FK506 production in the R2YE medium, recombinant strains showed strongly reduced FK506 titer in this medium. Similar results were obtained for industrially used M1 and M2 medium as well as for the ISPz for FK506 production. Since in our experiments only MGm medium delivered stable production levels, we performed all fermentations in this medium.

**FIGURE 3:**
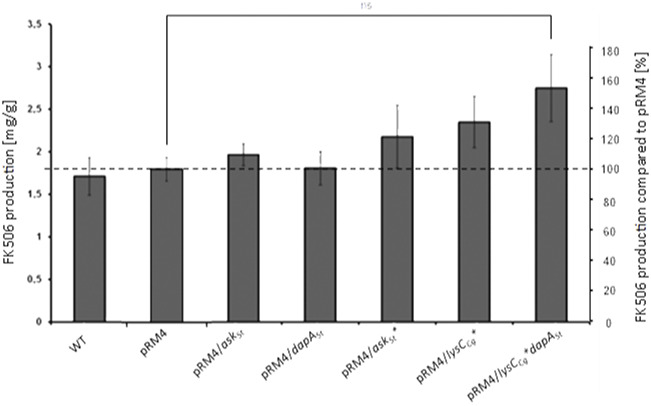
Comparison of the FK506 production of the wild type and the overexpression strains with optimized lysine supply. *Y*-axis on right: percentage representation of production optimization; as 100% the FK506 production of the wild type (WT) was set at the respective time. *Y*-axis on left: calculation of the mean of specific FK506 production over a fermentation period of 6 days. pRM4 stays for the integrative plasmid without an inserted gene. pRM4/–integration of an additional copy of the gene on the integrative plasmid pRM4 encoding: *askSt–*aspartate kinase; dapASt - dihydropicolinate synthase; askSt*—feedback insensitive aspartate kinase; lysCCg*—deregulated aspartate kinase from *C. glutamicum*.

Overexpression of both enzymes DapASt and AskSt in the wild type producer *S. tsukubaensis* NRRL18488 individually did not result in a significant increase of FK506 yield (Figure 3). Considering the feedback regulation of the aspartate kinase as the major bottleneck in lysine biosynthesis, it is not surprising that overexpression of the dihydropicolinate synthase (DapASt) alone does not lead to a profound effect on FK506 production (∼7%). The overexpression of AskSt alone demonstrated no significant effect on FK506 production (∼8%) (Figure 3), since this enzyme is subject to strict regulation by a final product inhibition lysine and threonine.

The primary strategy to relieve this bottleneck was constructing a feedback insensitive aspartate kinase (AskSt*) using the data of Sahm et al., 1996 available for the aspartate kinase from the actinomycete *C. glutamicum*. For this purpose a single amino acid exchange in the wild type Ask (from Ser301 to Tyr301) was introduced *via* site-directed mutagenesis. The resulting AskSt* was overexpressed in *S. tsukubaensis*. The growth of strains was monitored in parallel to the FK506 production measurement (Supplementary Figures S2, S3). The biomass dry weight estimation revealed almost identical growth profile of the tested strains in comparison to the WT. The overexpression of AskSt* in *S. tsukubaensis* lead to a slight enhancement of approximately 17% of FK506 production (Figure 3).

Therefore, a second strategy involving the heterologous expression of a gene encoding a deregulated aspartate kinase (AskCg* = LysCCg*) (Seibold et al., 2006) from the high-level lysine producer strain *C. glutamicum* ATCC 13032 has been used. In our approach, the askCg* gene was additionaly introduced into the genome of *S. tsukubaensis* on the integrative plasmid pRM4 under the control of the constitutively expressed ermEp promoter. This attempt resulted in a significant FK506 yield improvement of about 46% (Figure 3) delivering a more beneficial outcome than supplementation of the production medium with lysine only reaching 30% improvement (Figure 3). Combining overexpression of both enzymes, the deregulated aspartate kinase (LysCCg*) together with the dihydrodipicolinate synthase (DapASt), consistently shifted the carbon flux toward L-lysine formation and resulted in a considerable upgrade of FK506 production of about 73% (Figure 3). This demonstrates the advantage of genetic engineering in contrast to exogenous feeding approaches. The overexpression of both enzymes resulted in an overproduction of FK506, presumably from the increase of lysine availability. However, the increase in the lysine intracellular pool might not be a consequence of a redirection of the carbon flux, but a more efficient pathway. In cephamycin producing *S. clavuligerus* strain a positive correlation between deregulated aspartate kinases and the improvement of antibiotic production was also previously described (Özcengiz et al., 2010).

### Improving the pipecolic acid precursor supply in *Streptomyces tsukubaensis*

The essentiality of FkbL for FK506 production was tested by the construction of an *fkbL* deletion mutant in *S. tsukubaensis* through the replacement of *fkbL* by an apramycin resistance gene, which resulted in a tacrolimus null-mutant. Subsequently, the *fkbL* mutant was successfully complemented with *fkbL*. The FK506 production in the mutant and the recombinant strains grown in the MG production medium was analyzed by HPLC. The loss of FK506 production in the *ΔfkbL* mutant of *S. tsukubaensis* could also be reconstituted by exogenous addition of pipecolate, which again proved the indispensability of this building block for FK506 biosynthesis (Supplementary Figures S2, S3).

**FIGURE 4:**
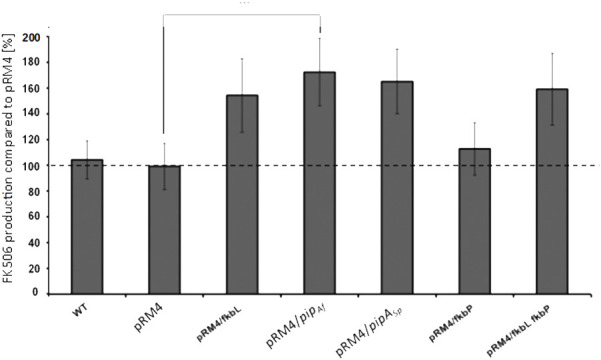
Comparison of the FK506 production in the *S. tsukubaensis* wild type with overexpression of different LCD genes. pRM4 stays for the integrative plasmid without an inserted gene. PRM4/–integration of an additional copy of the gene on the integrative plasmid pRM4 encoding: *fkbL*–LCD FkbL from *S. tsukubaensis*; *pipAf*–LCD PipAf from *A. friuliensis*; *pipASp*–LCD PipASp from *S. pristinaespiralis*; *fkbP*–NRPS FkbP. FK506 production was measured as an average from three replicates.

In order to test whether overexpression of the essential *fkbL* gene increases FK506 production, the plasmid pRM4/*fkbL* including the *fkbL* gene under control of the constitutive ermE* promoter was introduced into *S. tsukubaensis* WT. The resulting construct revealed a yield increase of 47%. (Figure 4).

Pipecolate is incorporated into the growing FK506 backbone by the NRPS FkbP, which could constitute a limiting factor for FK506 production. Therefore, the effect of overexpression of *fkbP* was analyzed. However, only a slight yield improvement (11%) was observed when *fkbP* alone was overexpressed. However, combination of overexpression of both genes *fkbL* and *fkbP* led to a final increase of ∼55% (Figure 4). These results were acquired from production assays, which were carried out in flask cultivation with the chemically defined medium. Similar observations were reported from fermentation studies with S. tsukubaensis D852, another FK506 producer (Huang et al., 2013). For this study, genes *fkbO*, *fkbL*, *fkbP*, *fkbM* and *fkbD* were introduced into the parental strain (each as a single construct) using the integrative *E. coli–Streptomyces* vector pIB139 containing the ermE* promoter (PermE*) and overexpressed.

Since apparently the production of pipecolate is a highly relevant criteria for efficient FK506 production, this step of the biosynthesis was analyzed in more detail. Pipecolic acid is a widely distributed constituent of secondary metabolites like for example the streptogramin antibiotic pristinamycin I from *Streptomyces pristinaespiralis* (Cocito, 1979; Mast et al., 2011) and the lipopeptide friulimicin B from *Actinoplanes friuliensis* (Aretz et al., 2000; Müller et al., 2007). In these microorganisms the biosynthesis of the pipecolate building block is also catalyzed by a lysine cyclodeaminase encoded by *pipASp* in *S. pristinaespiralis* (Mast et al., 2011) and by *pipAf* in *A. friuliensis* (Müller et al., 2007). The products of both genes, PipASr and PipAf, show a protein identity of 57% and 50% to the wild type FkbL, respectively. In order to test, whether the heterologous lysine cyclodeaminases can replace the endogenous enzyme, each of the lysine cyclodeaminase genes was expressed heterologously in the *ΔfkbL* mutant under the control of the constitutive ermE* promoter. Indeed PipASp as well as PipAf were able to restore FK506 production demonstrating that both enzymes are able to form pipecolic acid needed for FK506 biosynthesis (Supplementary Figures S2, S3). In order to compare the potential of the PipASp and PipAf for the improvement of FK506 we overexpressed both *pipASp* and *pipAf* in *S. tsukubaensis* wild type under the control of the constitutive ermE* promoter. An improvement of FK506 production of 56% in case of *pipASp* and 65% in case of *pipAf* overexpression was observed (Figure 4).

### Identification of structural features of the binding pocket in PipASp and its comparision with the model structures of PipAf and FkbL

Regarding the significant sequence homology of PipASp and PipAf to FkbL, it was rather unexpected to see a substantial difference in FK506 enhancement. So far, there was no evidence that the PipAf protein might be more efficient than the native FkbL in converting lysine into pipecolic acid. However, the results in FK506 enhancement were hinting towards structural differences and features of the binding pocket of PipAf that might allow it to be more efficient in lysine binding compared to PipASp and FkbL. To understand the basis for this effects, we aimed to analyze and compare the structures of the three lysine cyclodeaminases PipASp, PipAf and FkbL, to optimize subsequently the effectiveness of the FK506 production. Resolving of the LCD structure should provide a base for precise enzyme classification, understanding its properties, and future enzyme engineering.

The crystal structure of the LCD PipASp from *S. pristinaespiralis* was reported and deposited in the protein data base PDB as 5QZJ (Ying & Chen, 2016). Also, the biochemical activity of PipASp has previously been studied (Gatto et al., 2006; Tsotsou & Barbirato, 2007; Byun et al., 2015). The crystal structures of Pip from *A. friuliensis* as well as FkbL from *S. tsukubaensis* remained unknown so far. In order to compare the structural composition of PipASp, PipAf and FkbL, first homology modelling of PipAf and FkbL was performed. The structures of PipAf and FkbL were generated based on the template with the best query cover and known crystal structure–PipASp.

Although the PipASp structure has been deposited in the PDB database, its properties and key amino acids in the binding pocket have not been described. PipASp belongs to the µ-crystallin family and is related to ornithine cyclodeaminases (OCD). It is annotated as lysine decarboxylase WP_037775551.1, alongside with FkbL as WP_006350828.1 and other proteins including AF235504.1 in *S. hygroscopicus* (Wu et al., 2000) and AGP59511.1 in *S. rapamycinicus* (Baranasic et al., 2013). In contrast, PipAf is annotated in the NCBI database as OCD WP_023362365.1.

In order to identify possible key amino acids in the catalytic center of PipASp, we compared it with the structurally solved OCD from *Pseudomonas putida* PpOCD (Goodman at al., 2004) with PDB accession code 1X7D, using the Swiss-Model Structure Comparision tool and Swiss PDB-Viewer Magic Fit tool. PipASp consists, similar to described OCD, of a homodimeric fold whose subunits comprise two functional regions: a substrate-binding domain and a Rossmann fold that interacts with a dinucleotide positioned for re-hydride transfer (Supplementary Figure S4). In the OCD of P. putida, oligomerization results in a 14-stranded, closed β-barrel and each subunit contributes residues as shown in the Supplementary Figure S4) and Table 1. In the PipASp structure the β-barrel is similarly structured (Supplementary Figure S4, and Supplementary Figure S5; Table 1).

**TABLE 1:** Comparision of key amino acid residues in the catalytic domain of the ornithine cyclodeaminase PpOCD (1X7D) (Goodman et al., 2004), and the lysine cyclodeaminase PipASp (5QZJ) (Ying, 2016), as well as the models of PipAf, model of FkbL and the crystal structure of PipAf (this work).

Enzyme	Pp OCD	LCD PipAsp	LCD PipA, (model)	LCD FkbL	LCD pipm (structure)
PDBentry	1X7D	5Q13	none	none	this work
13-barrel	Phe4	Va15	Leu5	11e5	—
13-barrel	Tyr66	Trp64	Pro63/His65	Phe64	Trp61/Pro63/Glu60/Met62
13-barrel	Phe68	Leu76	Leu73	Met76	Lys74
13-barrel	Tyr70	Thr78	Leu75	Thr78	Tyr78
13-barrel	Phe88	Thr97	Thr94	Thr97	11e91/Tyr98
13-barrel	Tyr98	Ala107	His104	Ser107	—
13-barrel	Pro99	Leu108	Leu105	Leu108	Arg118
13-barrel	Trp325	His333	Leu330	Thr333	—
substrate carboxyl group side chains	Arg45	Arg49	Pro44/Pro45	Arg49	Arg46/Phe49
substrate carboxyl group side chains	Lys69	Lys77	Lys74	Lys77	—
substrate carboxyl group side chains	Arg112	Arg121	Arg118	Arg121	—
Ammonia leaving group side chains	Asp228	A1a235	Asp233	Ala235	As p233

**FIGURE 5:**
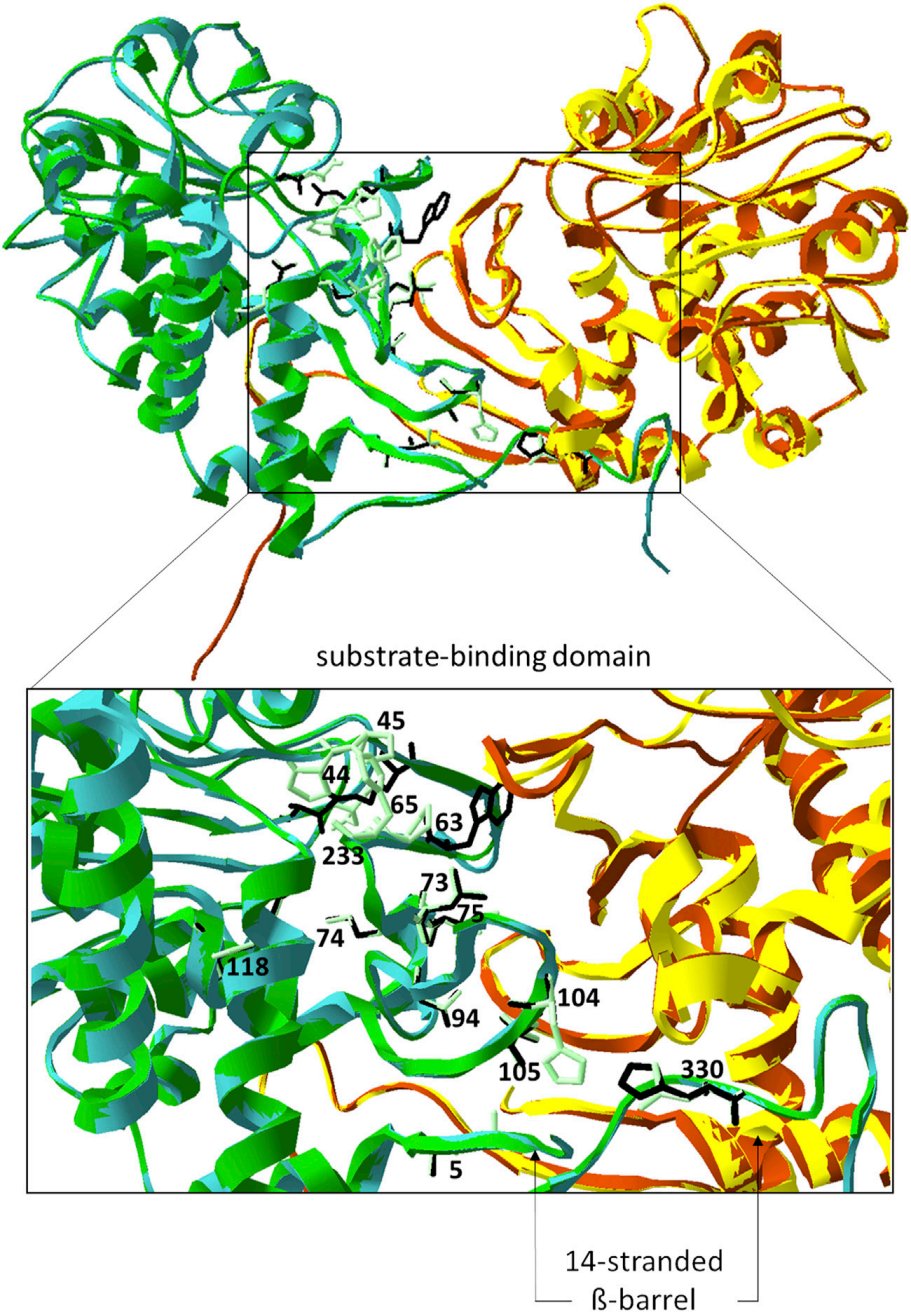
Model structure of PipAf. The dimer is depicted in green (first monomer) and yellow (second monomer)) superposed with the crystal structure of the lysine cyclodeaminase PipASp (the dimer in cyan (first monomer) and brown (second monomer)). Amino acid residues of PipAf marked in light green and labeled, of PipASp in black and non-labeled. Amino acid residues are demonstrated on one monomer. Zoom-in represents the substrate binding domain with the β-barrel (7-stranded on each monomer).

Based on the crystal structures of PpOCD and PipASp, the model structures of other LCDs PipAf and FkbL were generated through homology modelling. The structures of PipASp, PipAf and FkbL were overlaid; and the identified amino acid residues of PipASp were used to find the corresponding key amino acids in the catalytic center of PipAf and FkbL. Comparisons with PipASp demonstrated that in the lysine cyclodeaminase PipAf from *A. friuliensis* (Figure 5) the β-barrel is similarly but not identically structured (Supplementary Figure S5; Table 1). In the lysine cyclodeaminase FkbL from *S. tsukubaensis* the composition β-barrel differs from PipAf, but is almost identical to PipASp (Supplementary Figure S6; Table 1).

Our studies of the crystal structure of the lysine cyclodeaminase PipASp from *S. pristinaespiralis* as well as model structures of FkbL from *S. tsukubaensis* and PipAf from *A. friuliensis* revealed structural differences between PipAf and PipASt/FkbL. Key amino acids in PipAf and FkbL models were identified showing that PipAf features different amino acid residues in the catalytic domain (Figure 5; Table 1).

### Determination of the crystal structure of PipAf from *A. friuliensis* and its structural analysis

**FIGURE 6:**
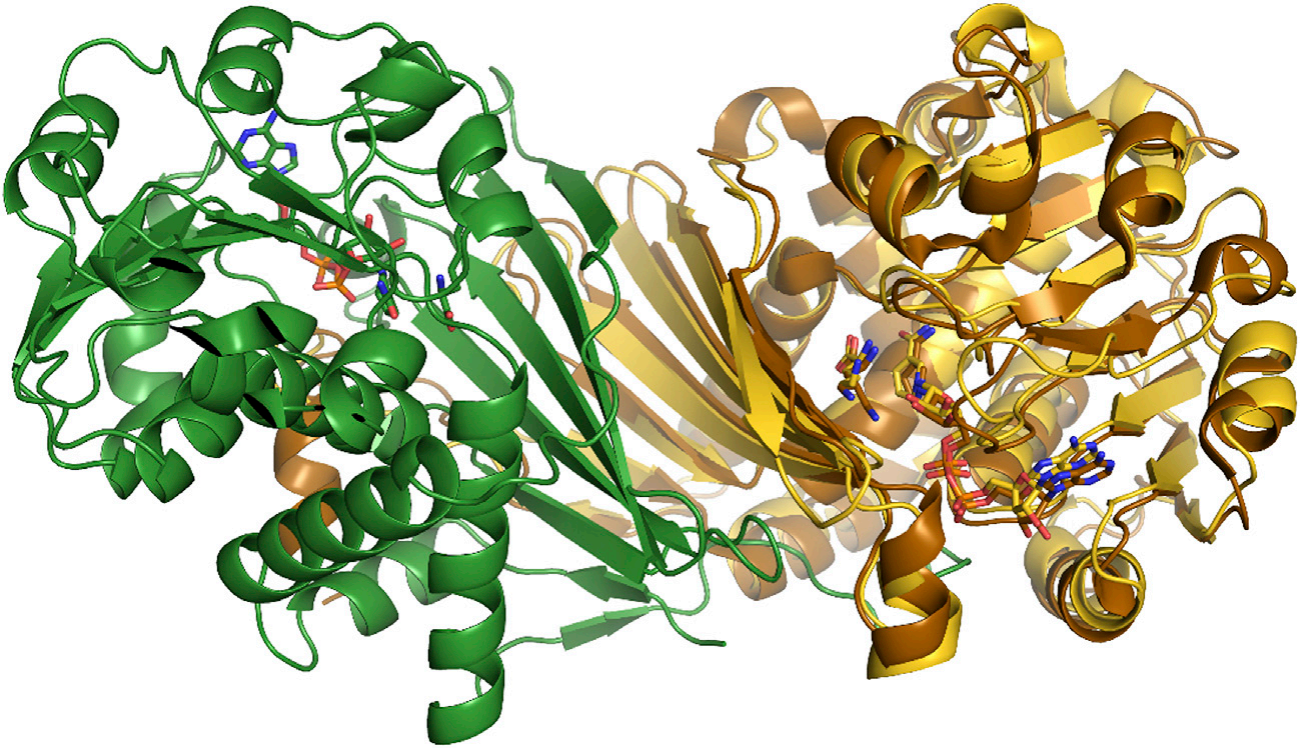
PipAf with NADH and lysine (dimer in yellow and green) superposed with OCD with NADH and ornithine (brown).

Homology modelling demonstrated that the composition of PipAf differs from PipASp and FkbL. In order to allow the engineering and specific design of PipAf for optimized FK506 production, we aimed to solve its crystal structure. PipAf was overexpressed as a His-tagged protein in *E. coli* BL21 and purified for further analysis. A monomer of PipAf consists of 339 residues and the purified enzyme appeared as a ∼36 kDa protein in SDS-page. Its oligomeric state from size exclusion chromatography was found to be dimeric as observed for many members of the µ-crystallin/OCD family, e.g. PpOCD (Goodman et al., 2004). PipAf crystals grew within few hours and diffracted up to 1.3 Å resulting in excellent crystallographic statistics (Supplementary Table S1). The enzyme consists of a homodimeric fold (Figure 6) whose subunits include two domains that function in the L-lysine and NAD+/H cofactor binding (substrate binding domain) as well as in oligomerisation of subunits. One molecule NADH binds per monomer *via* a canonical Rossman fold, consistent with previous reports for the structurally characterized PpOCD (Goodman et al., 2004; Dzurov et al., 2015).

A 7-stranded β-sheet domain forms on the one side an extended dimer interface of the PipAf LCD, on the other side it contributes most of the residues of the substrate binding pocket (Figure 6). The large interface between two monomer subunits with a surface of 2,996 Å^2^ confers the stability of the dimer with -45 kcal/mol as calculated by the PISA server. Interestingly, both subunits contribute alternating hydrophobic residues (Phe49, Trp61, Pro63 and Tyr98) to the dimer interface and lock it similar to zipper-folds.

The model of PipAf was compared with the obtained crystal structure of PipAf. The analysis of amino acid residues of interest in the catalytic site of the enzyme revealed that residues Pro63 and Asp233 could be confirmed *via* crystallographic studies to be involved into the key functions of the catalytic domain of the enzyme.

### Analysis of lysine binding pockets in the substrate binding domain of PipAf

**FIGURE 7:**
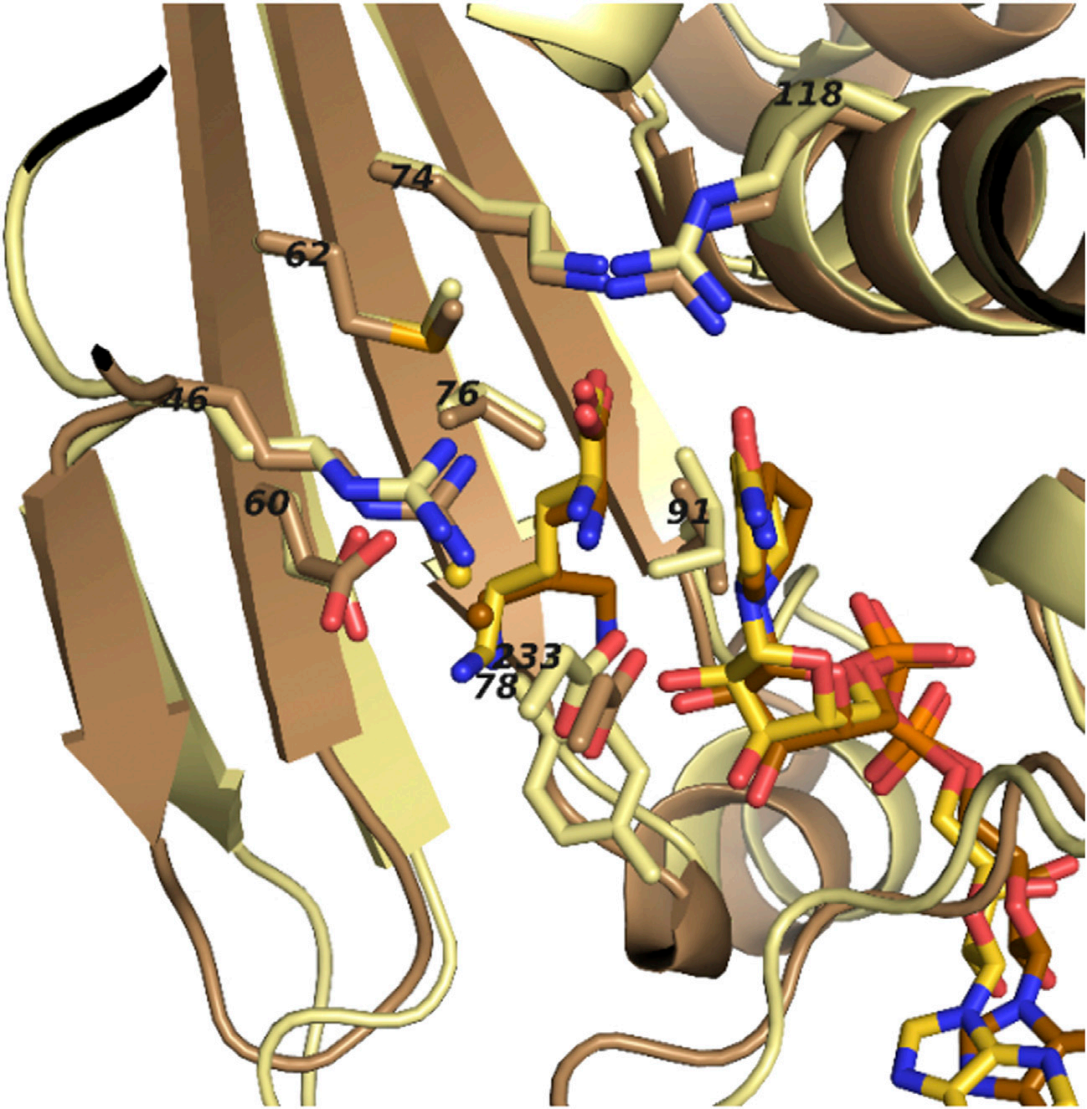
Superposed ligand binding pockets of PipAf with NADH and lysine (yellow) and PpOCD with NADH and ornithine (brown). Residue numbers refer to PipAf.

In order to enhance the properties of PipAf we were aiming to engineer the enzyme by site-directed mutagenesis. In order to identify relevant amino acids involved in substrate binding, PpOCD crystal structure was compared with the obtained PipAf crystal structure. The substrate binding domain contributes conserved residues essential for conversion of L-lysine to L-pipecolate and directs them closely to NAD+. Like in PpOCD, series of the acidic and basic residues interacting with lysine are highly conserved in PipAf (Arg46, Glu60, Met62, Lys74, Tyr78, Ile91, Arg118, Asp233). The domain is composed of a varied α/ β fold involving a 7-stranded β-sheet packed against three α helices. Lysine binds in the active site running parallel to the nicotinamide ring. Tyrosine 78 (Tyr78) and isoleucine 91 (Ile91) in the PipAf structure substitute smaller, but also hydrophobic residues glycine and valine in PpOCD respectively. Such residues should allow the lysine binding (Figures 6, 7).

### Analysis of dinucleotide binding pocket in the substrate binding domain of PipAf

Our attempts of PipAf engineering require in-depth understanding of the enzyme-dinucleotide binding kinetics. The catalytic mechanism of PipAf includes the dinucleotide cofactor binding at first, followed by substrate binding in an ordered reaction mechanism (Supplementary Figure S7). All reported dinucleotide folds, also in PpOCD, bind the pyrophosphoryl moiety of the dinucleotide *via* a glycine-rich, 30 to 35 amino-acids long loop comprising of a hydrophobic core, a negatively charged residue at the C-terminus and a positively charged residue at the N-terminus (Kleiger & Eisenberg, 2002; Goodman et al., 2004). A glycine-rich phosphate-binding sequence GxGxxG/A/S (where x is any residue) was found in all members of the µ-cristalline family, including PpOCD (Goodman et al., 2004). Whereas most phosphate-binding motifs have the sequence GxGxxG/A, OCDs exhibit the sequence GxGxxS (Goodman et al., 2004). This explains why previous studies dismissed the existence of canonical dinucleotide binding sequences in OCDs (Kim et al., 1992). A precedent for use of the GxGxxS sequence was established in studies of dihydropyrimidine dehydrogenase, which uses the latter motif in FAD+ binding (Dobritzsch et al., 2001). The C-terminal part of PipAf lacks the coil-α6 segment reported to contribute some residues to NAD+/H recognition in OCD and thereby distinguish LCD from the rest of the μ-crystallin family (Goodman et al., 2004).

After purification by affinity chromatography and size exclusion chromatography PipAf exhibited an absorption at 340 nm corresponding to at least one molecule of NADH per dimer, if calculated with the extinction coefficient for NADH in aqueous solution. Crystallization was always performed with 1 mM NAD+, but each PipAf seems to contain NADH as cofactor as indicated by the non-planarity nicotinamide ring, which was clearly visible due to the high resolution. In analogy to the proposed mechanism of OCD the catalytic cycle of PipAf should start with a NAD+ oxidizing the αalpha-amino group of lysine. The resulting imine group is substituted by the epsilon-amino group of lysine and the circular imine is finally reduced to pipicolic acid, thereby regenerating the NAD+ cofactor. OCD also contained NADH, therefore we would like to propose in this study an alternative 2-step mechanism for cyclodeamination in which a direct substitution of the alpha-amino group by the hydride ion from NADH takes place which in turn is substituted by the epsilon-amino group, based on findings described previously by Min et al., 2018.

### Site directed mutagenesis of key amino acids of PipAf to enhance the production of pipecolic acid

**FIGURE 8:**
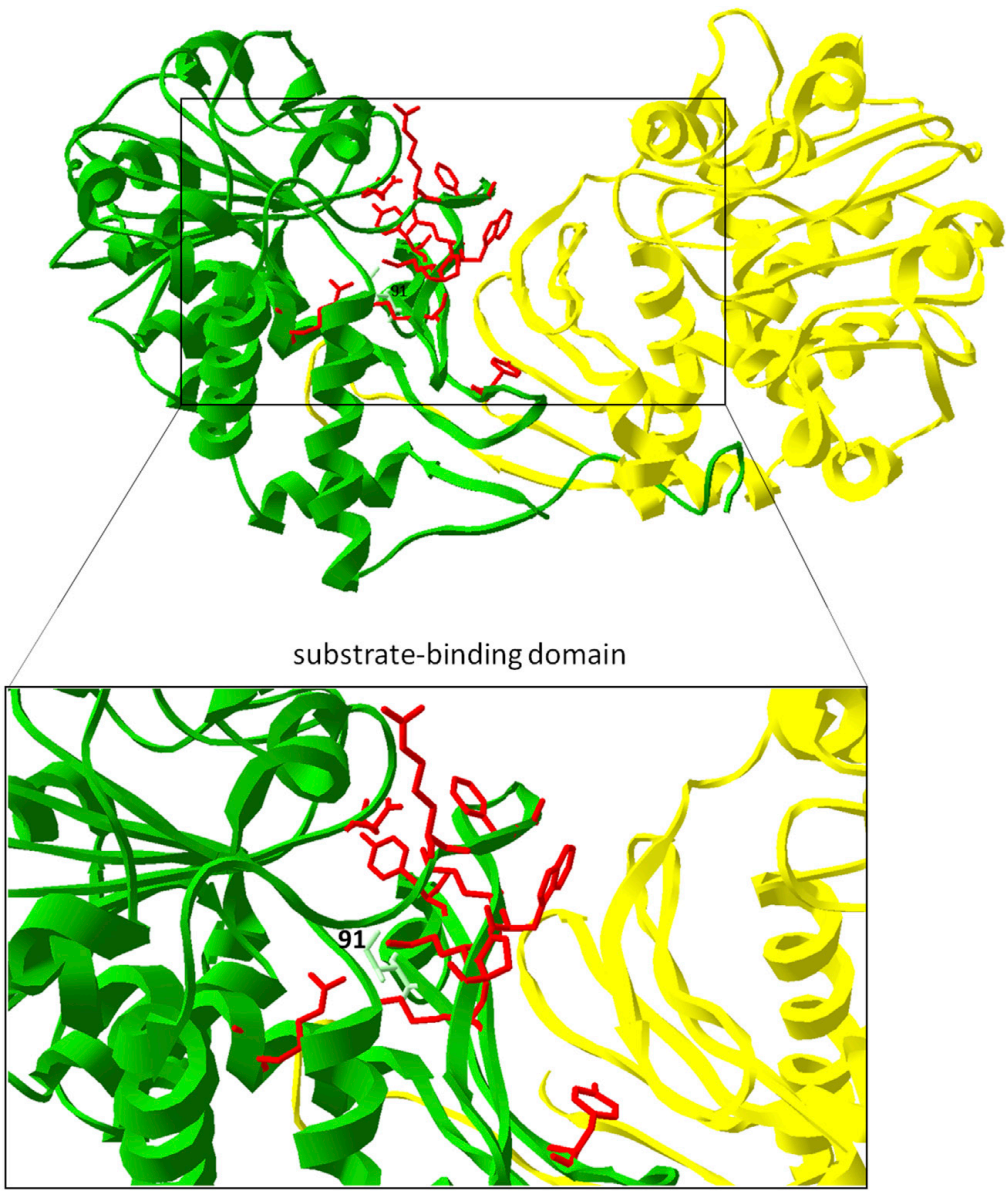
Engineering of PipAf. Key amino acid residues in the substrate-binding domain of PipAf are marked in red; the replacement of Ile91 by Val is marked in light green and labeled. The dimer is shown in green (first monomer) and yellow (second monomer). Amino acid residues are demonstrated on one dimer. Zoom-in represents the substrate binding domain (7-stranded β-barrel on each monomer).

In order to optimize the one-step LCD-mediated synthesis of pipecolic acid through metabolic engineering we aimed to carry out site-directed mutagenesis based on our structural studies. The mutations included the exchange of amino acid residues in the substrate binding domain, potentially leading to enhanced substrate binding capacities, including binding pocket residues: Val58 by Leu58, Val58 by Ala58, Glu60 by Ala60, Glu60 by Gln60, Glu60 by Leu60 (Figure 8, red; Table 1). The replacement of Val58 by Leu58 or Ala58 would lead to the strengthening or attenuation of the formation of a bond to the lysine side chain, which could be beneficial for deprotonation. The replacement Glu60 by Ala60 or Leu60 might open the entrance to the binding pocket a little more for the substrate. The replacement Glu60 by Gln60 might result in the generation of another hydrogen bond. Another strategy was the exchange of amino acid residues in the ammonia leaving group side chain residue Asp233 by Asn233 (Figure 8, red; Table 1). Such substitution might lead to the generation of another hydrogen bond as well. Especially interesting should be the exchange of the lysine binding residue Ile91 by Val91. Ile at this place corresponds to the Val in FkbL. IIle binds hydrophobically the middle part of lysine, unlike Val. In order to avoid the reduction in activity due to this fact, Ile91 was replaced through Val. (Figure 8, light green).

The selected amino acids were exchanged by site-specific mutagenesis in the pET30/pip expression plasmid. Using specifically designed primers with mutated bases, the mutagenesis PCR was carried out. The degradation of the non-mutated template DNA was performed with the restriction enzyme DpnI. Afterwards, the competent *E. coli* Novablue strain was transformed. Plasmid-carrying clones were identified by selection for kanamycin and verifyed by sequencing.

Mutated versions of the *pipAf* gene (pip*Ile91Val, pip*Val58Leu, pip*Val58Ala) were cloned into the expression vector and introduced into *E. coli* Rosetta 2 (DE3) pLysS. The product of the lysine cyclodeaminase reaction was detected *via* thin layer chromatography (TLC). In order to investigate and compare the activity of the individual mutants of the PipAf enzyme, a qualitative detection system for pipecolate production was established. The native pip gene from *A. friuliensis* served as reference. Since the host *E. coli* Rosetta 2 (DE3) pLysS has no lysine cyclodeaminase, the strain is not able to synthesize pipecolic acid and was therefore used as a negative control.

**FIGURE 9:**
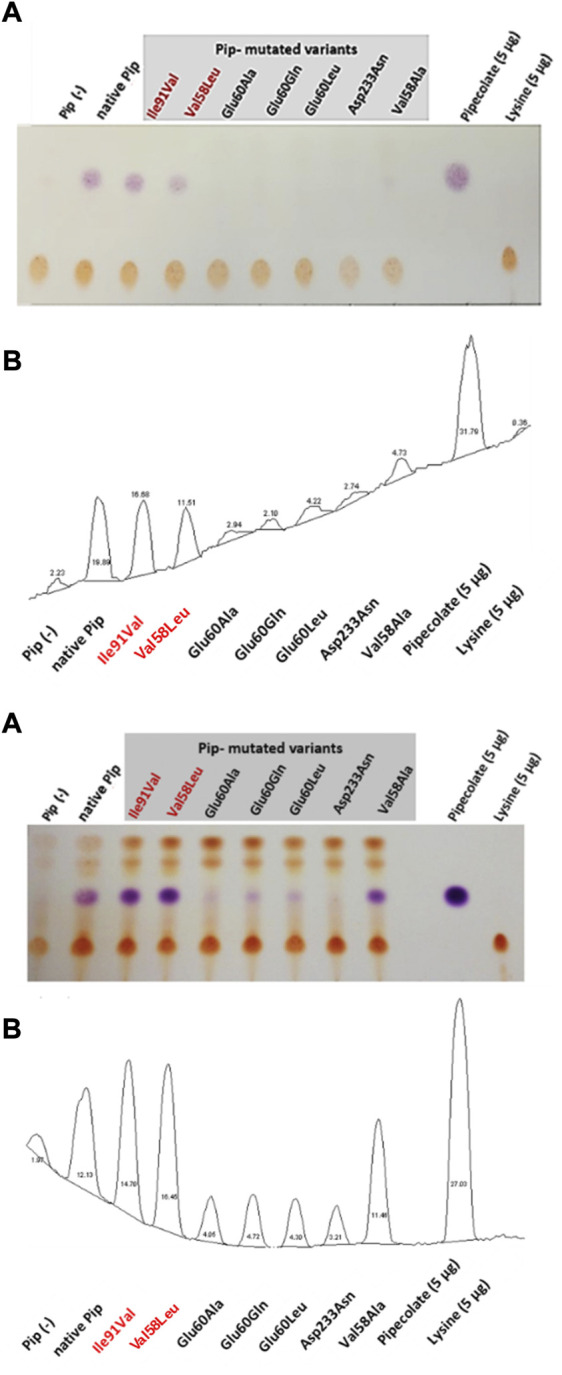
Proof of the pipecolate production in recombinant E. *coli* from two independent experiments. Sample injection: 5 µL of the culture supernatant. **(A)** Scan of the TLC plate (contrast optimized). Violet drops represent pipecolate, yellow dots: lysine. Mutants with the best pipecolate production are marked in red: Ile91Val and Val58Leu. **(B)** Relative quantification of pipecolate and lysine based on digital imaging (ImageJ). Percentage of each peak is related to the total area of all peaks. Each peak corresponds to the respective spot on the TLC plate.

TLC revealed that lysine could only be converted into pipecolic acid by the *E.coli* Rosetta strains carrying the plasmids pET30/pip*Ile91Val, pET30/pip*Val58Leu, pET30/pip*Val58Ala and the native pET30/*pip* with the native pipAf gene (Figure 9). In the mutated Pip variants Pip*Glu60Ala, Pip*Glu60Gln and Pip*Glu60Leu only a minimal pipecolic acid production was detected. In the case of the Pip* variant Pip*Asp233Asn, no pipecolic acid formation was observed. In the case of the Pip* variants Pip*Ile91Val and Pip*Val58Leu, the highest pipecolic acid formation was determined (Figure 9).

### FK506 production by metabolically engineered strains

**FIGURE 10:**
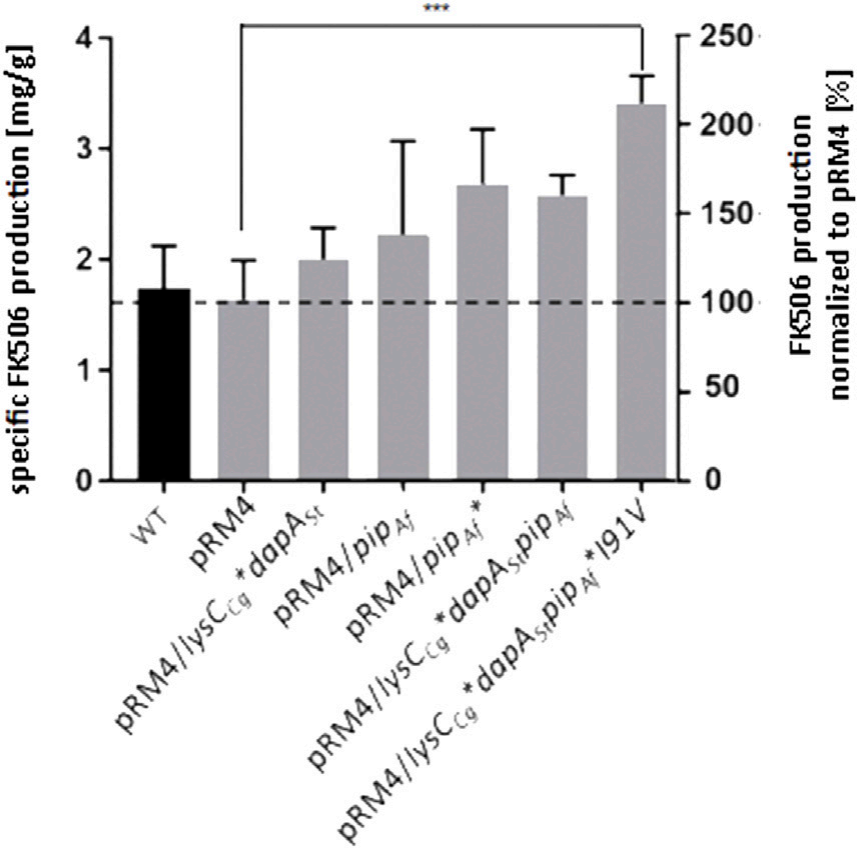
Production of FK506 in the *S. tsukubaensis* as well as in engineered strains. *Y*-axis on right: percentage representation of production optimization; as 100% the FK506 production of the wild type (WT) was set at the respective time. *Y*-axis on left: calculation of the mean of specific tacrolimus production over a fermentation period of 6 days. pRM4 stays for the integrative plasmid without an inserted gene. pRM4/ - integration of an additional copy of the gene on the integrative plasmide pRM4 encoding: *dapASt* - dihydropicolinate synthase; lysCCg*—deregulated aspartate kinase; *pipAf*–LCD from *A. friuliensis*; pipAf*I91V–LCD from *A. friuliensis* with Ile91 exchanged to Val. *p*-value: 0.0007.

According to the crystal structure of PipAf, Val91 does not hydrophobically bind the middle part of lysine, which should lead to enhanced enzyme activity. Thus, the mutated variant of Pip*Ile91Val was selected for the introduction into the recombinant host. The plasmid carrying the mutation pET30/pip*Ile91Val was generated by site-directed mutagenesis through the introduction of the point mutation using specific primers. Ile in contrast to Val should bind the middle part of the lysine hydrophobically, because of the different behaviour of their side-chains. *S. tsukubaensis* with the previously introduced *dapASt* and *lysCCg* genes was used as host. The specific FK506 production was quantified using RP-HPLC and represented in mg/g cells and in percentage. While the *S. tsukubaensis* WT could achieve a FK506 production of 1.7 mg/g cells, the S. tsukubaensis strain with the introduced mutated variant of PipAf produced of 3.4 mg/g, which is about 2-fold higher compared to the WT strain (Figure 10). Again, lysine supplementation did not result in an FK506 yield increase in the engineered strain *S. tsukubaensis* pET30/pip*Ile91Val, which correlates with studies showing that the availability of nutrients like lysine can have an inhibitory effect on antibiotic production (van Wezel and McDowall, 2011).

## Conclusion

The complex biosynthesis of FK506 in the wild type producer *S. tsukubaensis* only results in rather low titers. However, this biosynthesis includes key steps that can be open to attack. We could show that engineering two steps of FK506 production are suitable targets for optimization: the provision of lysine which is required as precursor for the FK506 building block pipecolate, and the conversion of lysine into pipecolate.

In order to increase the lysine pool, engineering of two reactions of the primary metabolism turned out to be successful: on the one hand directing the metabolic flux from aspartate to lysine was possible by overexpression of the dihydropicolinate synthase gene. On the other hand a synthetic biology approach using the gene for a feedback deregulated aspartate kinase from a lysine overproducing C. glutamicum resulted in a yield improvement.

The lysine converting lysine cyclodeaminase (LCD) was shown to be essential for generating pipecolate. Overexpression of the *S. tsukubaensis* LCD gene as well as that of LCD genes from other actinomycetes, which synthetize pipecolate-containing natural products, delivered recombinant *S. tsukubaensis* strains with increased FK506 yields. Since the different LCDs provided different levels of optimization, we concluded that metabolic engineering of LCD may offer an additional option for further increase. Indeed, after crystallization of the LCD from *A. friuliensis* it was possible to identify amino acids, the exchange of which by site-directed mutagenesis resulted in improved FK506 production.

Whereas the individual engineering steps led to moderate yield increase, the combination of overexpression of biosynthetic genes with enzyme design and with a synthetic biology approach resulted in a duplication of the FK506 yield.

## Data Availability

The original contributions presented in the study are included in the article/Supplementary Material, further inquiries can be directed to the corresponding authors.
