# Supplementary material for: Optimization of the precursor supply for an enhanced FK506 production in Streptomyces tsukubaensis
Source: Front Bioeng Biotechnol. 2022 Nov 18;10:1067467. doi: 10.3389/fbioe.2022.1067467 (PMC9715595; doi:10.3389/fbioe.2022.1067467)
Supplement: Supplementary file 1 [file Presentation1.pdf]

Supplementary materials:

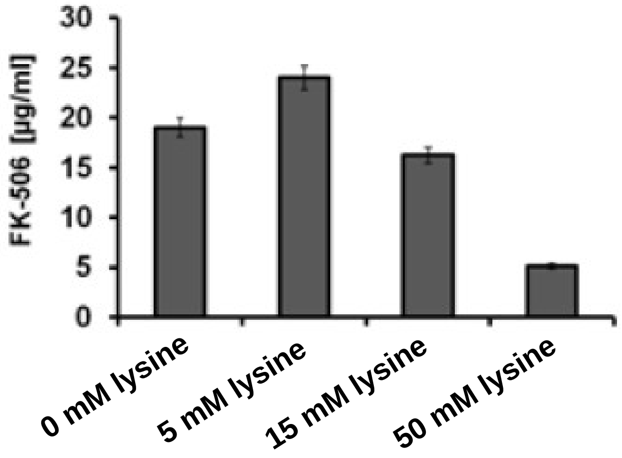

Supplementary Fig. 1. Production test of *S. tsukubaensis* WT in production medium MG with addition of different lysine concentrations. The exogenous supplementation of higher lysine concentrations leads to inhibited FK506 production.

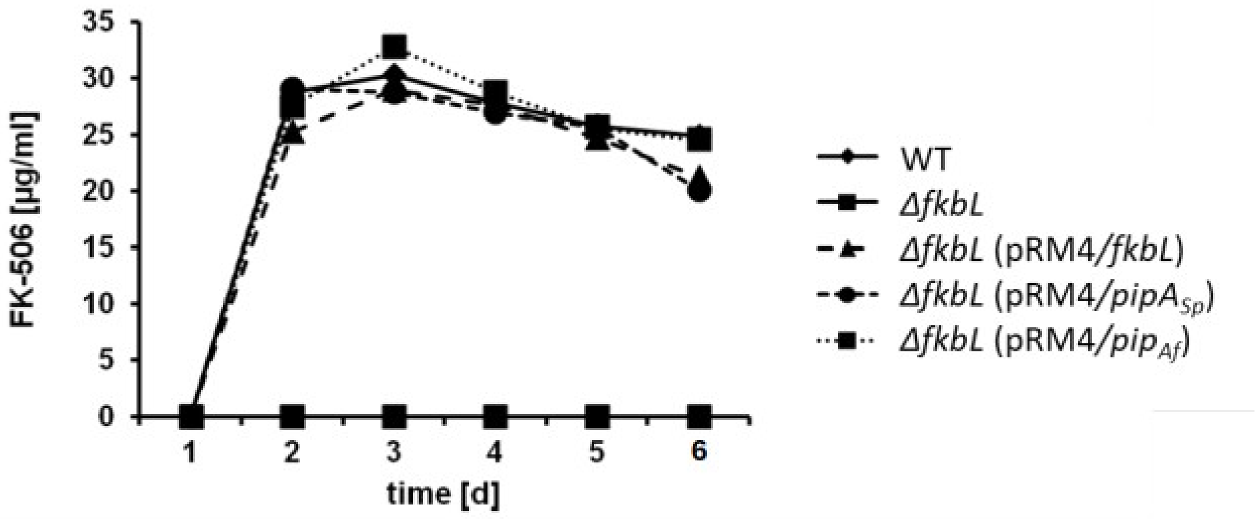

Supplementary Fig. 2. Genetic complementation of the Δ*fkbL S.tsukubaensis* mutant with lysine cylodeaminase genes from *S. tsukubaensis* WT, *S. pristinaespiralis* and *A. friuliensis.* each of the lysine cyclodeaminase genes was expressed heterologously in the Δ*fkbL* mutant under the control of the constitutive *ermE** promoter.

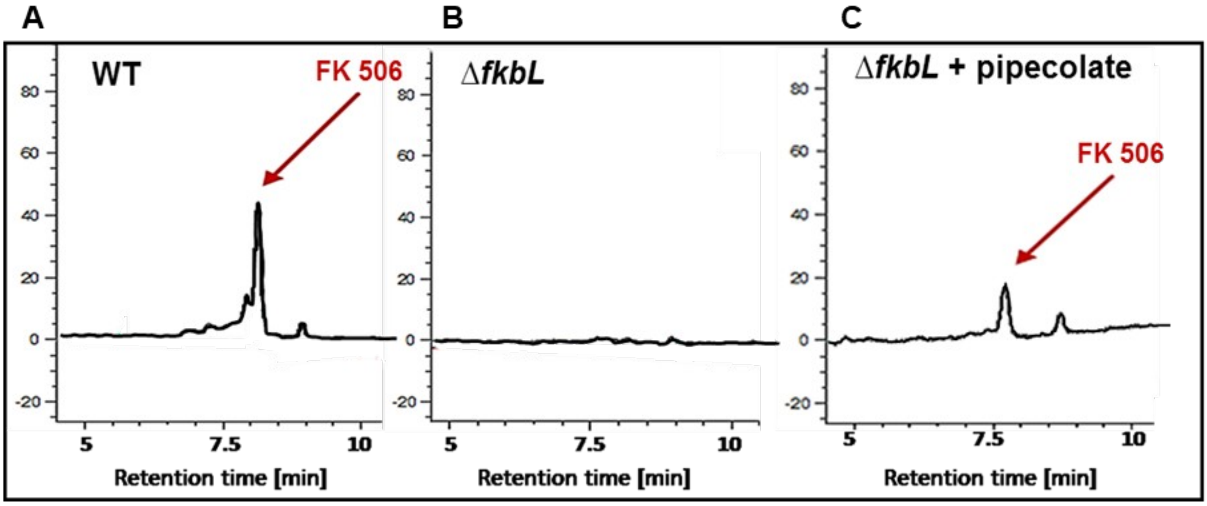

Supplementary Fig. 3. HPLC chromatograms for the detection of tacrolimus after three days of cultivation in MG medium. A) FK506 in the *S. tsukubaensis* WT. B) no FK-506 in the *S. tsukubaensis ΔfkbL* mutant. C) FK506 production in *S. tsukubaensis ΔfkbL* after the addition of pipecolate. Y-axis: absorbance in mAU, X-axis: retention time (minutes).

A

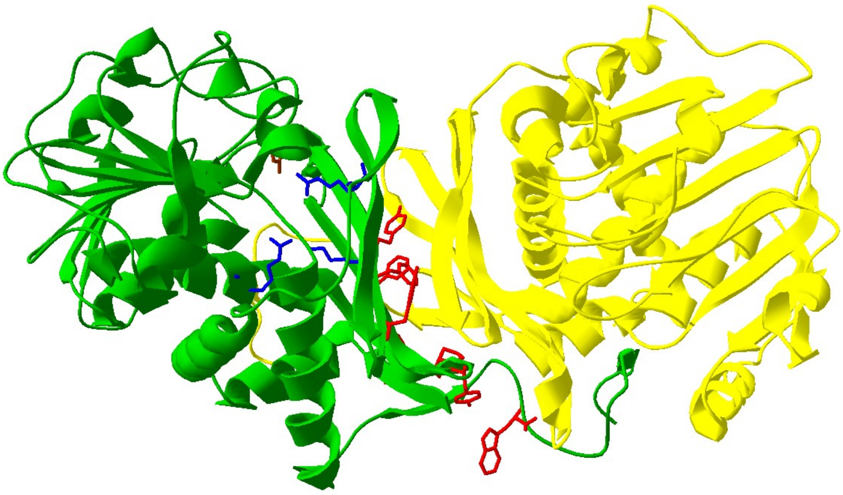

B

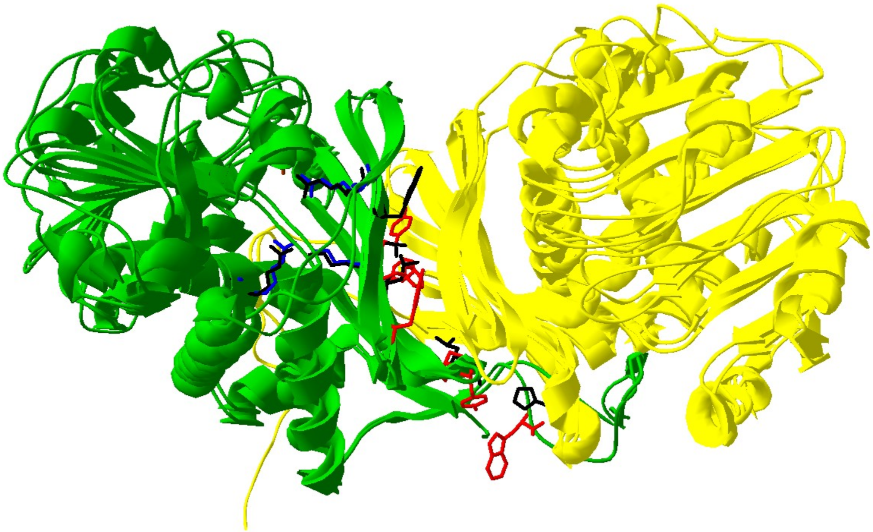

Suppl. Fig. 4. A. Structure of the ornithine cyclodeaminase from *Presudomonas putida* (1X7D), B. Comparision of the ornithine cyclodeaminase (1X7D) with the lysine cyclodeaminase from *Streptomyces pristinaespiralis* (5QZJ). Key amino acid residues are marked in red, blue and brown (1X7D) and black (5QZJ).

In the OCD of *P. putida* (1X7D), oligomerization results in a 14-stranded, closed β-barrel and each subunit contributes residues, namely Phe4, Tyr66, Phe68, Tyr70, Phe88, Tyr98, Pro99, and Trp325 (A, red) to the barrel interior in the substrate-binding domain. Moreover, the substrate carboxyl group interacts with the side chains of Arg45, Lys69, and Arg112 (A, blue) and the ammonia leaving group hydrogen bonds to the side chain of Asp228 (A, brown) (after Kim & Park, 2007; Goodman at al., 2004).

A

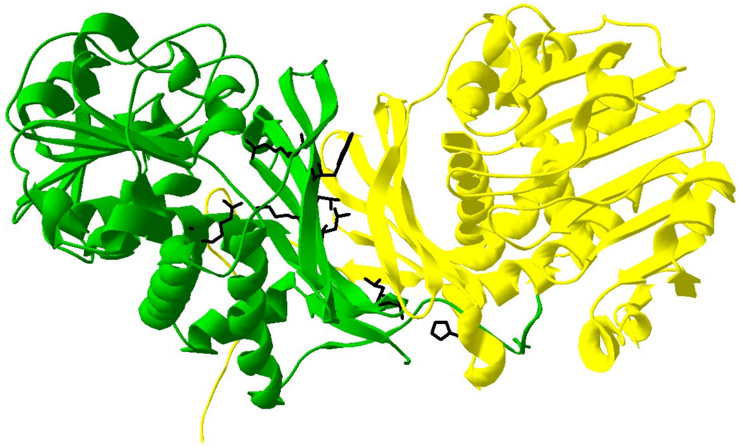

B

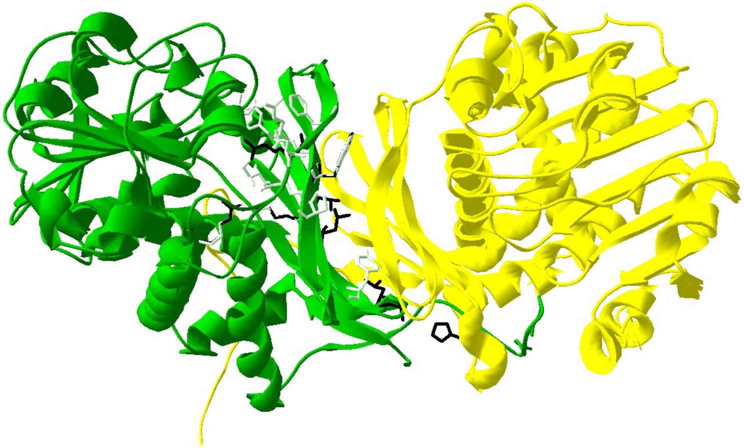

C

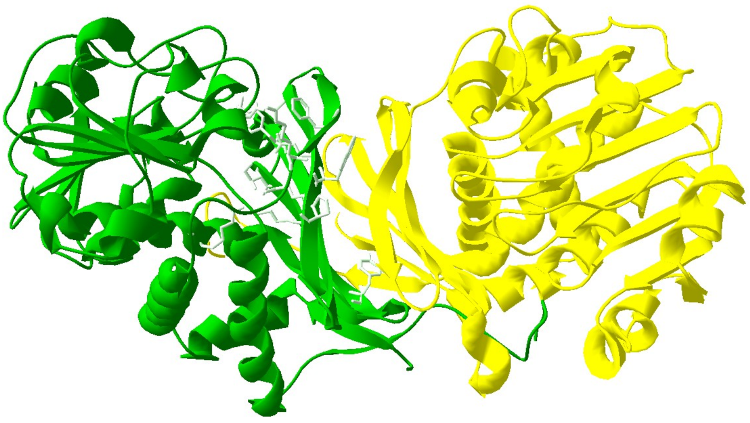

Suppl. Fig. 5. A. Structure of the lysine cyclodeaminase from *Streptomyces pristinaespiralis* (5QZJ); B. Comparision of the the lysine cyclodeaminase (5QZJ) with the model of the lysine cyclodeaminase Pip$_{Af}$, C. Model

of the lysine cyclodeaminase from *Actinoplanes friuliensis.* Key amino acid residues are marked in black (5QZJ) and green (Pip_{Af}).

In the $PipA_{Sp}$ structure (5QZJ) the β-barrel is similarly structured involving at the same place residues Val5, Trp64, Leu76, Thr78, Thr97, Ala107, Leu108 and His333 compared to 1X7D. The substrate carboxyl group side chains include Arg49, Lys77 and Arg121 and the side chain for the ammonia leaving group the residue Ala235 (Suppl. Fig. 4, B, black; Suppl. Fig. 5, A). Comparisons with $PipA_{Sp}$ demonstrated that in the lysine cyclodeaminase Pip_{Af} from *A. friuliensis* the β-barrel is similarly but not identically structured involving at the same place residues Leu5, Pro63/His65, Leu73, Leu75, Thr94, His104, Leu105, Leu330 (Suppl. Fig. 5, B, C, light green) compared to 5QZJ (Suppl. Fig. 3, A, B, black). The substrate carboxyl group side chains include Pro44/Pro45, Lys74, Arg118 and the side chain for the ammonia leaving group the residue Asp233 in the Pip_{Af} structure (Suppl. Fig. 5, B, C, light green).

A

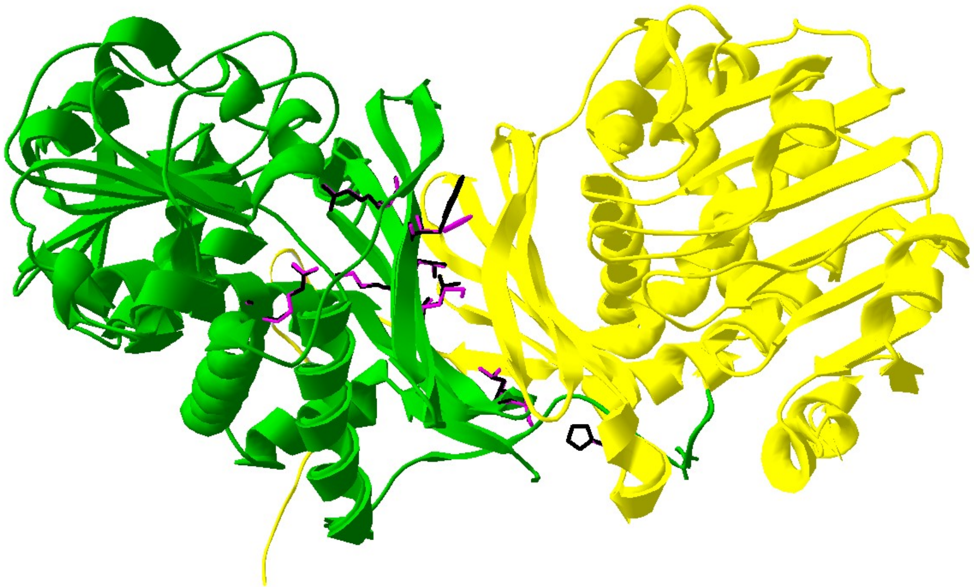

B

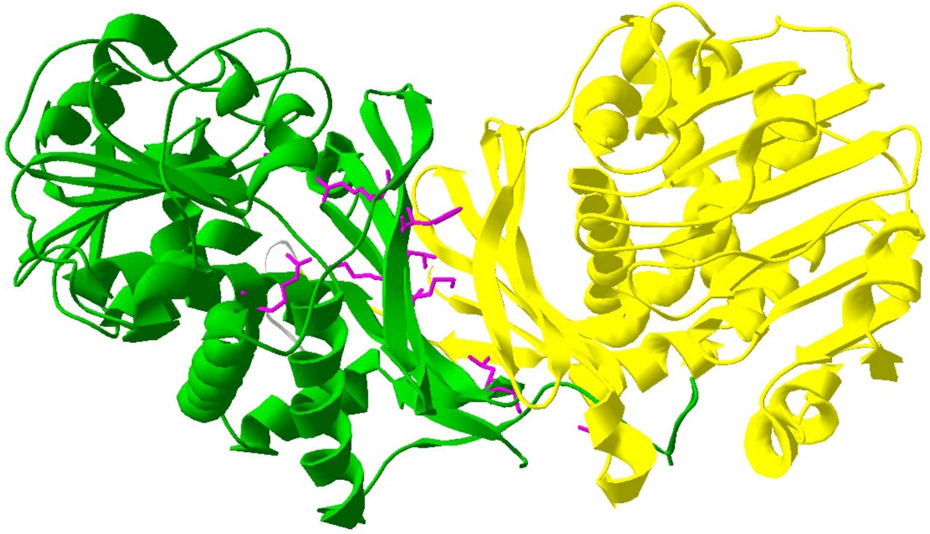

Suppl. Fig. 6. A. Comparision of the the lysine cyclodeaminase from *S. pristinaespiralis* (5QZJ) with the model of the lysine cyclodeaminase FkbL, B. Model of the lysine cyclodeaminase from *Streptomyces tsukubaensis.* Key amino acid residues are marked in black (5QZJ) and purple (FkbL).

In the lysine cyclodeaminase FkbL from *S. tsukubaensis* the composition β-barrel differs from Pip_{Af}, but is almost identical to $PipA_{Sp}$ involving at the same place residues Ile5, Phe64, Met76, Thr78, Thr97, Ser107, Leu108, Thr333 (Suppl. Fig. 6, A, B, purple) compared to 5QZJ (Suppl. Fig. 6, A, black). The substrate carboxyl group side chains include Arg49, Lys77, Arg121 and the side chain for the ammonia leaving group the residue Ala235 in the FkbL structure (Suppl. Fig. 6, A, B).

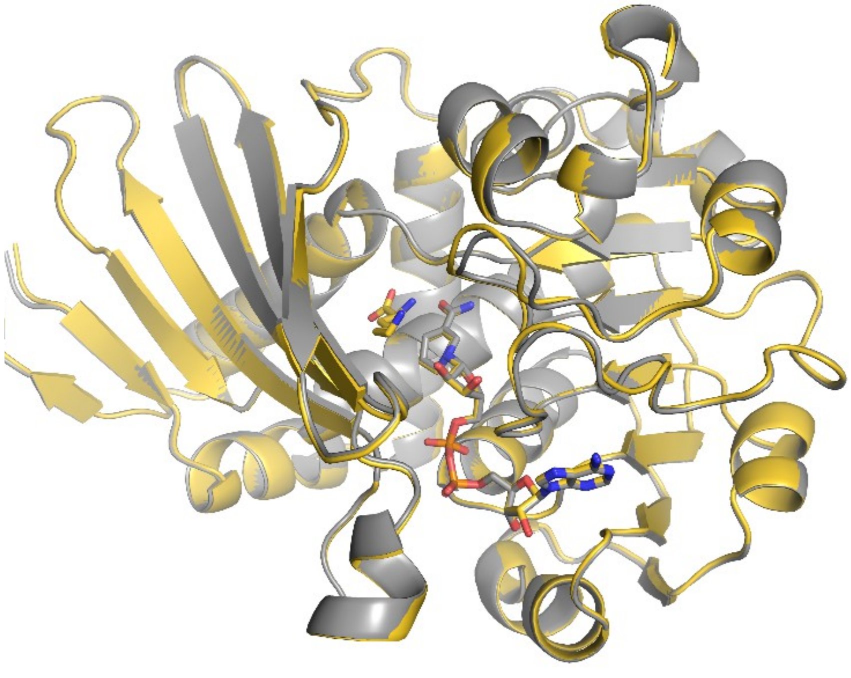

Suppl. Fig.7. Pip_{Af} (yellow) with NADH and lysine superposed with Pip_{Af} with only NADH (grey).

Data collection statistics

	Pip$_{Af}$	Pip$_{Af}$ complex with Lys
Beamline	SLS X06DA	SLS X06DA
Wavelength λ [Å]	1.000	1.000
Detector	Pilatus 2M	Pilatus 2M
Detector distance [mm]	135	120
Space group	$P2_12_12_1$	$P2_12_12_1$
Unit cell: [Å]<br>[degree]	59.2 89.9 137.3<br>90.0 90.0 90.0	59.5 89.2 139.1<br>90.0 90.0 90.0
Resolution [Å]	50 – 1.4 (1.49 – 1.4)	50.0 – 1.3 (1.38 – 1.3)
No. of reflections		
	1318869 (195149)	1306889 (186423)
	145265 (23022)	181827 (28151)
R_{meas} [%]	10.3 (153.4)	6.7 (112.7)
CC1/2	59.1	72.2
Completeness (%)	99.8 (98.8)	99.3 (96.0)
Multiplicity	9.1 (8.5)	7.2 (6.6)
$\langle I\rangle/\langle\sigma(I)\rangle$	12.1 (1.35)	14.5 (1.62)
Wilson Factor [Å^2]	24.2	22.9
Crystal Mosaicity [°]	0.076	0.083

Refinement statistics

	Pip$_{Af}$	Pip$_{Af}$ complex with Lys
Resolution range [Å]	50 – 1.3	50 – 1.4
R_{Cryst}	0.219	0.205
R_{free} (test set of 5%)	0.242	0.226
No. of non-H atoms (partial occupancy)		
	2552 / 2551	2552/2547
	88	88

	-	50
	212	205
Average isotropic B-Factor [Å^2]	20.7	22.8
	24.7 / 18.7	20.6 / 18.4
	26.9 / 21.2	23.3 / 20.9
	17.9	16.3
	-	21.2
	23.5	23.0
Rmsd for bond lengths [Å]	0.006	0.006
Rmsd for bond angle [°]	1.15	1.11
Ramachandran regions		
	97.9	97.9
	1.9	2.0
	0.1	0

Suppl. Table. 1: Crystallographic data collection and refinement statistics

Strains/Plasmids	Genotype/Phenotype	Reference
E. coli NovaBlue	*recA*1, *endA*1, *gyrA*96, *thi*-1, *hsdR*17 (rK12-,mK12+) *supE*44, *relA*1, *lac* [F', *pro*AB, *lacI*^{q}, *lacZ*ΔM15, Tn*10*] (Tet^R)	Novagen
E. coli BL21(DE3)pLysS	F-, *ompT*, *hsdS*B (rB-mB-), *gal*, *dcm*, (DE3), pLysS, Cam^R, pLysS	Novagen
E. coli Rosetta 2(DE3) pLysS	Derivate of BL21, pRARE2: 7 rare tR-NAs; rare *E. coli* codons: arginine (AGA, AGG, CGA) glycine (GGA), isoleucine (AUA), leucine (CUA), pro-line (CCC)	Novagen
E. coli ET12567/pUZ8002	Methylation deficient strain *E. coli* with pUZ8002, F-, *dam*- 13::Tn*9*, *dcm*-6, *hsdM*, *hsdR*, *lacY*1, Cam^R, Kan^R	MacNeil et al.1992

E. coli ET12567/pUB307	Methylation deficient strain *E. coli* with pUB307, F-, *dam*-13::Tn*9*, *dcm*-6, *hsdM*, *hsdR*, *lacY*1, Cam^R, Kan^R, Tet^R	Bennett et al., 1977; MacNeil et al., 1992
S. coelicolor M145	*S. coelicolor* A3(2) without native plasmids	Kieser et al., 2000
S. pristinaespiralis Pr11	Pristinamycin producer wild type	Aventis Pharma
Streptomyces tsukubaensis NRRL18488	STP1 STP2	Martinez-Castro et al., 2012
Streptomyces tsukubaensis *ΔfkbL*	Derivate of *S. tsukubensis* WT; fkbL replaced by Apr^R; deficient in FK506 production	This work
pRM4	pSET152$_p$*ermE* with artificial RBS, Apr^R	Menges et al., 2007
pRM4/*fkbL*	pRM4 Derivate with *fkbL* (from *S. tsukubaensis*), Apr^R	This work
pRM4/*fkbP*	pRM4 Derivate with *fkbP* (from *S. tsukubaensis*), Apr^R	This work
pRM4/*fkbL fkbP*	pRM4 Derivate with *fkbL*, *ermE* and *fkbP* (from *S. tsukubaensis*), Apr^R	This work
pRM4/*pipA*	pRM4 Derivate with *pipA* (from *S. pristinaespiralis*), Apr^R	This work
pRM4/*pip*	pRM4 Derivate with *pip* (from *A. friuliensis*), Apr^R	This work
pRM4/*pip* * E60A	pRM4 Derivate with *pip* * ^{E60A}, Apr^R	This work
pRM4/*pip* * I91V	pRM4 Derivate with *pip* * ^{I91V}, Apr^R	This work
pRM4/*pip* * D233N	pRM4 Derivate with *pip* * ^{D233N}, Apr^R	This work
pRM4/*pip* * L234A	pRM4 Derivate with *pip* * ^{L234A}, Apr^R	This work

pRM4 kan/*fkbL*	pRM4/*fkbL*-Derivate with additional Kan^R, Apr^R	This work
pRM4 kan/*pipA*	pRM4/*pipA*-Derivate with additional Kan^R, Apr^R	This work
pRM4 kan/*pip*	pRM4/*pip*-Derivate with additional Kan^R, Apr^R	This work
pRM4/*ask*	pRM4-Derivate with *ask* (from *S. tsukubaensis*), Apr^R	This work
pRM4/*ask* *	pRM4-Derivate with mutated *ask**S301Y (from *S. tsukubaensis*), Apr^R	This work
pRM4/*lysC* *	pRM4-Derivate with deregulated *lysC** (from *C. glutamicum*), Apr^R	This work
pRM4/*dapA*	pRM4-Derivate with *dapA* (from *S. tsukubaensis*), Apr^R	This work
pRM4/*dapA lysC* *	pRM4-Derivate with *dapA*, *ermE* und *lysC* *, Apr^R	This work
pSET152	pUC18 *lacZα*, *oriT* (RK2), RP4 *mob* region, ΦC31 *int* and attP, Apr^R	Bierman, et al., 1992; Schmitt-John & Engels, 1992
pDRIVE	T7 RNA-Polymerase Promotor, SP6 RNA-Polymerase promoter, pUC origin, phage f1 origin of replication, *lacZα*, Amp^R , Kan^R	Qiagen
pJET 1.2/blunt	rep (pMB1),T7 RNA-Polyerase promoter, modified P_{lac} promoter for expression of *eco47IR* and positive selection (PlacUV5), Amp^R	Fermentas
pK18	pUC-derived, *LacZ*’ α-complementa-	Pridmore et al., 1987

	tion system, (Kan^R) + *oriT*	
ΔpK18oriT*fkbL*apra	pk18-Derivate with the inactivation construct for *fkbL*, Kan^R	This work
pYT9	pJOE2775-Derivate, Amp^R	Tiffert et al., 2008
pET30 Ek/LIC	Ligation Independent Cloning (LIC), T7 promoter, T7 start of transcription, phage f1 origin of replication, N-terminal His-tag and S-tag, C-terminal His-tag, T7 terminator, *lacI* coding sequence, pBR322 *ori*, Kan^R	Novagen
pET30/*pip*	Derivate pET30 with *pip* (from *A. friuliensis*), Kan^R	This work
pET30/*pip* $^{*\ E60A}$	Derivate pET30 with *pip* $^{*\ E60A}$, Kan^R	This work
pET30/*pip* $^{*\ E60Q}$	Derivate pET30 with *pip* $^{*\ E60Q}$, Kan^R	This work
pET30/*pip* $^{*\ E60L}$	Derivate pET30 with *pip* $^{*\ E60L}$, Kan^R	This work
pET30/*pip* $^{*\ I91V}$	Derivate pET30 with *pip* $^{*\ I91V}$, Kan^R	This work
pET30/*pip* $^{*\ D233N}$	Derivate pET30 with *pip* $^{*\ D233N}$, Kan^R	This work
pET30/*pip* $^{*\ V58L}$	Derivate pET30 with *pip* $^{*\ V58L}$, Kan^R	This work
pET30/*pip* $^{*\ V58A}$	Derivate pET30 with *pip* $^{*\ V58A}$, Kan^R	This work

Suppl. Tab 2. Strains and plasmids used in this study.

Oligonucleotides	Sequences 5´-3´	Reference
pipAfwNdeI	CATATGATGGAGACCTGGGTCCTGG	This work

pipArevBglII	AGATCTTCAGTGGGCGGGGGC	This work
pipfwNdeI	CATATGATGGATACGCTCCTGCTGAC	This work
piprevEcoRI	GAATTCGGTCAGCTGTAGGGGTTGAG	This work
fkbLEX_fwNdeI	CATATGATGCAGACCAAGATCCTGCGTG	This work
fkbLEX_revBglII	AGATCTTCACCACGGCAGCGAGTAGG	This work
fkbPEX_NdeI	CATATGGTGACACCGGACGGCAAGAG	This work
fkbPEX_neuHindIII	AAGCTTCTACTCGCTTCCCACGG	This work
fkbLup_fw	TCTAGAGCCGGGAGGGCCAGCGC	This work
fkbLup_rew	CATATGGTGGTGACGCCGGCCGGG	This work
fkbLdown_neu_fw	TGATATCGAGCGTCGTGGTGGTG	This work
fkbLdown_neu_rew	AAGCTTCGGCGCAACACTCGATAC	This work
Apranewfw	TAACATATGGGAGGCCAAACGGCATTG	This work
Apranewrev	ACAGATATCGGCCCACAGAATGATGTCAC	This work
oriT_ A	GCTAGCGGCCTCCGACTAACGAAAAT	This work
oriT_B	GCTAGCTCTTTTCCGCTGCATAACCC	This work
dapAfw_NdeI	CATATGATGGCTCCGATCCCCACTC	This work
dapArev_BglII	AGATCTTCAGAGCTGGACGCCTCCG	This work
Kanfw_BlpI	AAGCTCAGCGCTTCACGCTGCCGCAAGCACTCA	This work
NeurevKanBlpI	TGCTGAGCAGGGGTGGGCGAAGAACTCCAGCAT	This work
lysC_fw_NdeI	CATATGGTGGGCCTTGTCGTGCAG	This work
lysC_rev_HindIII	AAGCTTTCATCGGCCGGTGCCTC	This work

LysCTyrfw	GAATGTGTACGCGGCCACCACCGCTCTGACCGAC	This work
LysCTyrrew	GGTGGCCGCGTACACATTCTGGACGATCATGTCCAG	This work
erme_lysC_Bam1	GGATCCTTAGCGTCCGGTGCCTG	This work
erme_lysC_Bam2	GGATCCCGCGTTGGCCGATTC	This work
pippETfw	GACGACGACAAGATGGATACGCTCCTGCTGAC	This work
PippETrew	GAGGAGAAGCCCGGTCAGCTGTAGGGGTTGAG	This work
Glu60Glnfw	GCGTCATCCAGTGGATGCCGCACC	This work
Glu60Glnrew	CATCCACTGGATGACGCCGGTGTCAC	This work
Glu60Leufw	GCGTCATCCTGTGGATGCCGCACC	This work
Glu60Leurew	CATCCACAGGATGACGCCGGTGTCAC	This work
Glu60Alafw	GCGTCATCGCCTGGATGCCGCACC	This work
Glu60Alarew	CATCCAGGCGATGACGCCGGTGTCAC	This work
Asp233Asnfw	CGGCGCCAACCTCGTCGGCAAGTTCG	This work
Asp233Asnrew	GACGAGGTTGGCGCCGATCGCGTTG	This work
Val58Leufw	GACACCGGCCTGATCGAGTGGATGC	This work
Val58Leurew	CACTCGATCAGGCCGGTGTCACCGGG	This work
Val58Alafw	GACACCGGCGCCATCGAGTGGATGC	This work
Val58Alarew	CACTCGATGGCGCCGGTGTCACCGGG	This work
Ile91Valfw	CCGACGGTCATCGGCACGCTGACC	This work

Ile91Valrew	CCGATGACCGTCGGCAGGTTGAGG	This work

Suppl. Tab. 3. Oligonucleotides used in this study
